# The third restriction–modification system from *Thermus aquaticus* YT-1: solving the riddle of two TaqII specificities

**DOI:** 10.1093/nar/gkx599

**Published:** 2017-07-12

**Authors:** Piotr M. Skowron, Brian P. Anton, Edyta Czajkowska, Joanna Zebrowska, Ewa Sulecka, Daria Krefft, Joanna Jezewska-Frackowiak, Olga Zolnierkiewicz, Malgorzata Witkowska, Richard D. Morgan, Geoffrey G. Wilson, Alexey Fomenkov, Richard J. Roberts, Agnieszka Zylicz-Stachula

**Affiliations:** 1 Department of Molecular Biotechnology, Faculty of Chemistry, University of Gdansk, Wita Stwosza 63, 80-308 Gdansk, Poland; 2 New England Biolabs, 240 County Road, Ipswich, MA 01938, USA

## Abstract

Two restriction–modification systems have been previously discovered in *Thermus aquaticus* YT-1. TaqI is a 263-amino acid (aa) Type IIP restriction enzyme that recognizes and cleaves within the symmetric sequence 5′-TCGA-3′. TaqII, in contrast, is a 1105-aa Type IIC restriction-and-modification enzyme, one of a family of *Thermus* homologs. TaqII was originally reported to recognize two different asymmetric sequences: 5′-GACCGA-3′ and 5′-CACCCA-3′. We previously cloned the *taqIIRM* gene, purified the recombinant protein from *Escherichia coli*, and showed that TaqII recognizes the 5′-GACCGA-3′ sequence only. Here, we report the discovery, isolation, and characterization of TaqIII, the third R–M system from *T. aquaticus* YT-1. TaqIII is a 1101-aa Type IIC/IIL enzyme and recognizes the 5′-CACCCA-3′ sequence previously attributed to TaqII. The cleavage site is 11/9 nucleotides downstream of the A residue. The enzyme exhibits striking biochemical similarity to TaqII. The 93% identity between their aa sequences suggests that they have a common evolutionary origin. The genes are located on two separate plasmids, and are probably paralogs or pseudoparalogs. Putative positions and aa that specify DNA recognition were identified and recognition motifs for 6 uncharacterized *Thermus*-family enzymes were predicted.

## INTRODUCTION

The Type IIC/IIG restriction–modification (R–M) systems, in which the restriction endonuclease (REase) and methyltransferase (MTase) are part of the same polypeptide, are diverse and widely distributed in nature (1,2). They account for more than a third of all Type II R–M systems (2). Type IIC enzymes are γ-amino-MTases with N-terminal DNA-cleavage domains, able to cleave DNA at fixed distances from their recognition sequences (1). These compact systems are overabundant in mobile genetic elements (MGEs), especially in plasmids (2).

Two large families of closely related Type IIC/IIG enzymes have been described and broadly investigated: the MmeI-family (3–6) and the *Thermus* enzyme family (7–19). Both families recognize and methylate one strand of asymmetric sequences and cleave at a fixed distance downstream of the recognition site. MmeI-family enzymes cleave 21/19 nucleotides (nt) downstream from the adenine that is methylated, while *Thermus*-family enzymes cleave 11/9 nt downstream (8,14). The specificity of MmeI has successfully been altered at positions 3, 4 and 6 of the asymmetric 5′-TCCRAC-3′ DNA recognition sequence, resulting in several new DNA specificities (5,6). The *Thermus*-family enzymes, like Type I REases and the MmeI family, have features that make them naturally adapted to generate recognition sequence diversity.

The *Thermus*-family has been divided in two related subfamilies: (a) the TspGWI subfamily, which includes thermostable TaqII (10,13,16,19,20) and TspGWI (7,9,11,15) and (b) the TspDTI subfamily, which includes thermostable TspDTI (8,12), TsoI (12,14,18), and the non-identical isoschizomer pair TthHB27I/Tth111II (12,17,21,22). Moreover, several putative proteins from mesophilic or thermophilic bacteria found in GenBank (https://www.ncbi.nlm.nih.gov/genbank/, 23), also belong to the family, providing a suitable platform for specific DNA recognition studies and rational protein engineering (14,18,22). The existence of such homologous systems in distantly related species suggests frequent horizontal gene transfer (HGT) despite environmental isolation of thermophilic bacteria.

One of the most interesting members of the *Thermus*-family enzymes is TaqII (10,13,16,19,20). The protein was isolated from the thermophilic bacterium *T. aquaticus* YT-1 (20), a strain that also includes the Type II R–M system TaqI, which recognizes 5′-TCGA-3′. The crude preparation of TaqII described in the original study had two major activities, which recognized and cleaved 5′-GACCGA-3′ and 5′-CACCCA-3′ DNA sequences, respectively, and an additional minor contaminating REase activity with unknown recognition sequence (20). Our previous studies revealed that recombinant TaqII is prone to classic ‘star activity’, caused by pH, salt or enzyme concentration changes and exhibits the unique ‘affinity star’ specificity induced by sinefungin (SIN) (10,13). Although this could explain the minor contaminating activity (20), the existence of an additional R-M system could not be excluded.

Our preliminary research confirmed that the two major activities were not separated when TaqII was prepared from the native host. However, we discovered that recombinant TaqII isolated from the mesophilic bacterium *Escherichia coli* (*E. coli*), recognized only the 5′-GACCGA-3′ sequence (10). Such an unusual change of DNA substrate recognition specificity could be caused by number of factors, including: (*i*) a difference in protein tertiary or quaternary structure, (*ii*) different polypeptide folding in *E. coli*, (*iii*) posttranslational modifications specific for the genus *Thermus*, (*iv*) requirement for specific *Thermus* chaperonins, (*v*) an additional TaqII subunit missing from the recombinant enzyme complex or (*vi*) the existence of a third R–M system.

In the present study, we designed and performed experiments necessary to test the last one of the proposed hypotheses, namely that a second Type IIC enzyme was present in the native TaqII preparation. As a result we identified and sequenced a paralogous *taqIIIRM* gene and discovered novel cleavage specificity: TaqIII, a (nonidentical) twin enzyme of TaqII.

## MATERIALS AND METHODS

### Bacterial strains, plasmids, media and reagents

A recombinant plasmid expressing TaqII (pRZ-wt-*taqIIRM*) was previously constructed (16). Bacteriophage lambda DNA was obtained from Vivantis (Subang Jaya, Malaysia). Q5 DNA Polymerase, BsaI, XbaI, BspHI and SalI were from New England Biolabs (Ipswich, MA, USA). PCR primers were from Sigma-Aldrich (St Louis, MO, USA). Sanger DNA sequencing was conducted at GATC Biotech AG (Cologne, Germany). Miniprep DNA Purification Kit, T4 DNA Ligase, pBR322, pUC19 DNA and protein markers were from Thermo Fisher Scientific (Fermentas, Vilnus, Lithuania). Genomic DNA purification kit was from Qiagen (Germantown, MD, USA). Marathon DNA Polymerase was from A&A Biotechnology (Gdynia, Polska). *T. aquaticus* YT-1 was purchased from the DSMZ culture collection (Braunschweig, Germany). The bacteria were cultivated in a modified Lysogeny Broth (LB) [0.5% tryptose, 0.3% yeast extract, 0.2% NaCl, 0.001% dilution of 2.1 g/l stock Nitsch's trace elements, pH 7.2]. *E. coli* Top10 {F^−^*mcrA Δ(mrr-hsdRMS-mcrBC) ϕ80lacZΔM15 ΔlacX74 nupG recA1 araD139* Δ *(ara-leu)7697 galE15 galK16 rpsL(Str^R^) endA1* λ^−^} (Invitrogen, Carlsbad, CA, USA) or Endura^TM^ {*recA13 supE44 ara-14 galK2 lacY1 proA2 rpsL20(Str^R^) xyl-5 λ^–^ leu mtl-1 F^–^ mcrB mrr hsdS20(r_B_^–^, m_B_^–^)* (Lucigen Corp., Middleton, USA) were used for plasmid DNA purification. *E. coli* BL21 Star^TM^ (DE3) {F^–^*ompT hsdS_B_ (r_B_^−^, m_B_^−^) gal dcm rne*131 λ (DE3)} (Thermo Fisher Scientific (Invitrogen)) was employed for *taqIIRM* and *taqIIIRM* gene expression. The bacteria were cultivated in LB or Terrific Broth (TB) medium (24). Media were supplemented with chloramphenicol (CM) (40 μg/ml) and 0.2% maltose. BCIP/NBT solution was from BioShop Canada Inc. (Ontario, Canada). 6 ml Resource™ Q and Resource™ S ion exchange columns were from GE Healthcare Life Sciences (Uppsala, Sweden). All other reagents were purchased from Sigma-Aldrich (St Louis, MO, USA).

### Purification of native TaqII and TaqIII

*T. aquaticus* YT-1 bacteria were cultivated in a biofermentor Bioflo 310 (New Brunswick Scientific, Edison, NJ, USA) as described in Supplementary data.

The purification scheme for native TaqII/TaqIII from *T. aquaticus* YT-1 included the stages: cell lysis, DEAE-cellulose chromatography, precipitation of acidic proteins/nucleic acids with polyethyleneimine (PEI) (25), TaqII/TaqIII extraction from nucleic acid-acidic proteins-PEI complexes, ammonium sulfate (AmS) precipitation, Resource Q chromatography, Resource S chromatography and Heparin–Agarose affinity chromatography. The detailed purification procedure is provided in Supplementary data.

After Resource S chromatography two enzyme preparations were obtained: (*i*) fraction A cleaving only 5′-CACCCA-3′, which did not bind to the cation exchanger and (*ii*) fraction B, which eluted at ∼550 mM NaCl and exhibited REase activity against both 5′-GACCGA-3′ and 5′-CACCCA-3′. Both enzyme preparations were desalted and further purified to homogeneity using Heparin–Agarose (Supplementary data).

### DNA cleavage assays

A series of new 390 bp PCR DNA substrates amplified from pBR322, containing 5′-GACCGA-3′ and/or 5′-CACCCA-3′, were created using an approach described previously (10) (Supplementary data, Figure S1).

For detection of REase activity in chromatographic fractions a 390 bp PCR substrate containing convergently arranged 5′-GACCGA-3′ and 5′-CACCCA-3′ DNA recognition sequences (→←) was used (Supplementary data, Figure S1).

For determination of the TaqIII recognition sequence and cleavage site, various DNA substrates were used: the 390 bp PCR products, pUC19, pBR322 and λ DNA.

All TaqIII cleavage reactions were performed and analyzed as described in Supplementary data.

### Determination of TaqIII recognition sequence and cleavage site

#### Sanger sequencing

For sequencing, a 497 bp substrate was prepared as described previously (10). The purified restriction fragments were sequenced as described in Supplementary data.

### Western blotting

Purified proteins were separated by SDS-PAGE and electroblotted onto a PVDF membrane (24). The membrane was probed with rabbit anti-TaqII antibodies (courtesy of Dr D. Nidzworski, University of Gdansk, Poland), washed with TBS-T buffer and incubated with anti-rabbit secondary antibody conjugated with alkaline phosphatase (Santa Cruz Biotechnology, courtesy of Dr D. Nidzworski, University of Gdansk, Poland). A specific protein was visualized by adding BCIP/NBT solution. The detailed procedure is provided in Supplementary data.

### LC–MS–MS/MS analysis

LC–MS–MS/MS analysis (liquid chromatography coupled to tandem mass spectrometry) were performed at a Mass Spectrometry Laboratory (IBB PAS, Warsaw, Poland) as described in Supplementary data. The raw data were processed using Mascot Distiller followed by Mascot Search (Matrix Science, UK) against the predicted TaqII derived reference peptide masses. The search parameters are provided in Supplementary data. Peptides with a Mascot Score exceeding the 5% False Positive Rate threshold and with a Mascot Score exceeding 30 were considered to be positively identified.

### *T. aquaticus* YT-1 genome sequencing and assembly

*T. aquaticus* YT-1 total DNA was purified from a freshly grown liquid culture using the DNeasy Blood and Tissue Kit (Qiagen, Germantown, MD, USA) with minor modifications. DNA was sheared to an average size of 10 kb using G-Tubes (Covarys, Woburn, MA, USA). The sheared DNA (consisting of both chromosome and plasmids) was sequenced using the Single-Molecule Real-Time (SMRT) Sequencing platform (Pacific Biosciences, Menlo Park, CA, USA). Two libraries (‘SMRTbells’) were constructed as recommended by the manufacturer using the 20 kb library preparation protocol. Libraries were quantified and analysed using a Qubit fluorimeter (Invitrogen, Eugene, OR, USA) and a 2100 Bioanalyzer (Agilent Technology, Santa Clara, CA, USA). Both libraries had mean insert lengths of 10 kb. A portion of one library was size-selected for SMRTbells larger than 6 kb using a BluePippin device (Sage Science, Beverly, MA, USA).

The libraries were sequenced on an RSII instrument with C4-P6 chemistry and 240 min movie collection times (Pacific Biosciences, Menlo Park, CA, USA). Data from four SMRT cells (1.6 Gb sequence from two non-size-selected cells and 1.2 Gb from two size-selected cells), was used in the final analysis, resulting in mean chromosomal coverage of ∼680×. Reads were processed, mapped, and assembled *de novo* using the SMRT analysis pipeline with the HGAP.3 protocol. Closure and final assemblies were performed with the assistance of BLAST (26) and RS_BridgeMapper.1 from the SMRT Analysis suite (Pacific Biosciences, Menlo Park, CA, USA). Methylation analysis and determination of methylated motifs (27,28) were performed using RS_Modification_and_Motif_analysis.1 from the same suite.

### Cloning of the *taqIIIRM* gene

#### PCR amplification

Due to the high similarity between *taqIIRM* and *taqIIIRM* genes, *taqIIIRM* was amplified from *T. aquaticus* YT-1 plasmid DNA using a nested PCR approach (Supplementary data).

#### DNA cloning

The 3326 bp PCR fragment was cleaved with BspHI and SalI, subjected to agarose electrophoresis, gel isolated and cloned into the modified pRZ4737 vector (9), containing the P_R_ promoter under the control of the CI repressor (Supplemenary data).

#### Selection of positive bacterial clones

Both BsaI and BspHI/SalI cleavage of plasmid DNA as well as direct PCR from a single bacterial colony were used for the screening of clones. Plasmids from positive clones were subjected to DNA sequencing. The promoter regions and the *taqIIIRM* gene sequences of the recombinant plasmids were confirmed.

#### Recombinant gene expression

The *taqIIIRM* gene expression was performed in *E. coli* BL21 Star™ (DE3), as described in Supplementary data. Bacterial clones efficiently expressing *taqIIIRM* gene were selected for a large-scale bacterial culture.

### Purification of recombinant TaqII and TaqIII

The purification scheme for recombinant TaqII or TaqIII from *E. coli* included the following stages: cell lysis and heat treatment, precipitation of acidic proteins with PEI, TaqII/TaqIII extraction from nucleic acid-acidic proteins-PEI complexes, 20–40% AmS fractionation and size exclusion chromatography. The detailed purification procedure is provided in Supplementary data.

### Bioinformatics

Bioinformatic characterization of the nt sequences of pTAYT1_11 and pTAYT1_61 was performed using specialized software tools. The sequences were initially analysed using Geneious version 9.1.6 (29), ORF Finder (http://www.ncbi.nlm.nih.gov/gorf/gorf.html), SnapGene (http://www.snapgene.com), DNA Plotter (30) and Artemis (31). Similarity searches were performed using the BLAST programs (https:/blast.ncbi.nlm.nih.gov/, 26) and REBASE (32). Sequence alignments were performed using PROMALS3D (33). Putative promoter sequences were predicted using BPROM (http://www.softberry.com/berry.html) and PePPER (http://pepper.molgenrug.nl) (34).

### Nucleotide and protein sequence accession numbers

The nt sequences of plasmids pTAYT1_11 and pTAYT1_61 determined in this study have been annotated and deposited in GenBank with the accession numbers CP020571 and CP020572 respectively.

## RESULTS

### Cation exchange chromatography resulted in partial separation of two TaqII specificities

The initial characterization of TaqII by Barker *et al*. showed that a preparation of the enzyme from *T. aquaticus* specifically recognized two DNA sequence variants: 5′-GACCGA-3′ and 5′-CACCCA-3′ (20). In our previous work, we demonstrated that recombinant TaqII was able to recognize only 5′-GACCGA-3′ (10). We hypothesized therefore that the protein preparation obtained by Barker and co-workers was a mixture of two REases, TaqII and a hypothetical enzyme TaqIII that recognized only the 5′-CACCCA-3′ site.

**Figure 1.**
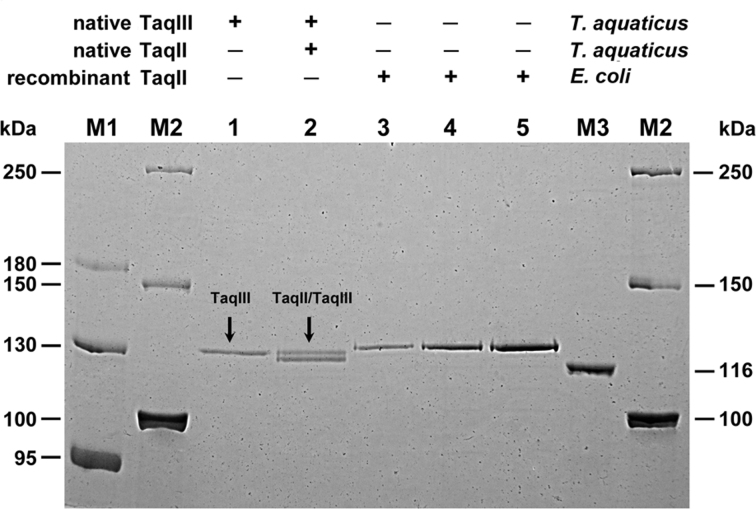
SDS-PAGE analysis of the purified native TaqII and TaqIII in a 6% gel. Lane M1, protein marker (Thermo Fisher Scientific), bands marked: 180, 130, 95 kDa; lane M2, protein marker (Thermo Fisher Scientific), bands marked: 250, 150, 100 kDa; lane 1, purified, homogeneous native TaqIII protein; lane 2, mixture of native TaqII/TaqIII proteins; lanes 3–5, different amounts of recombinant TaqII; M3, protein marker (Thermo Fisher Scientific), band marked: 116 kDa.

**Figure 2.**
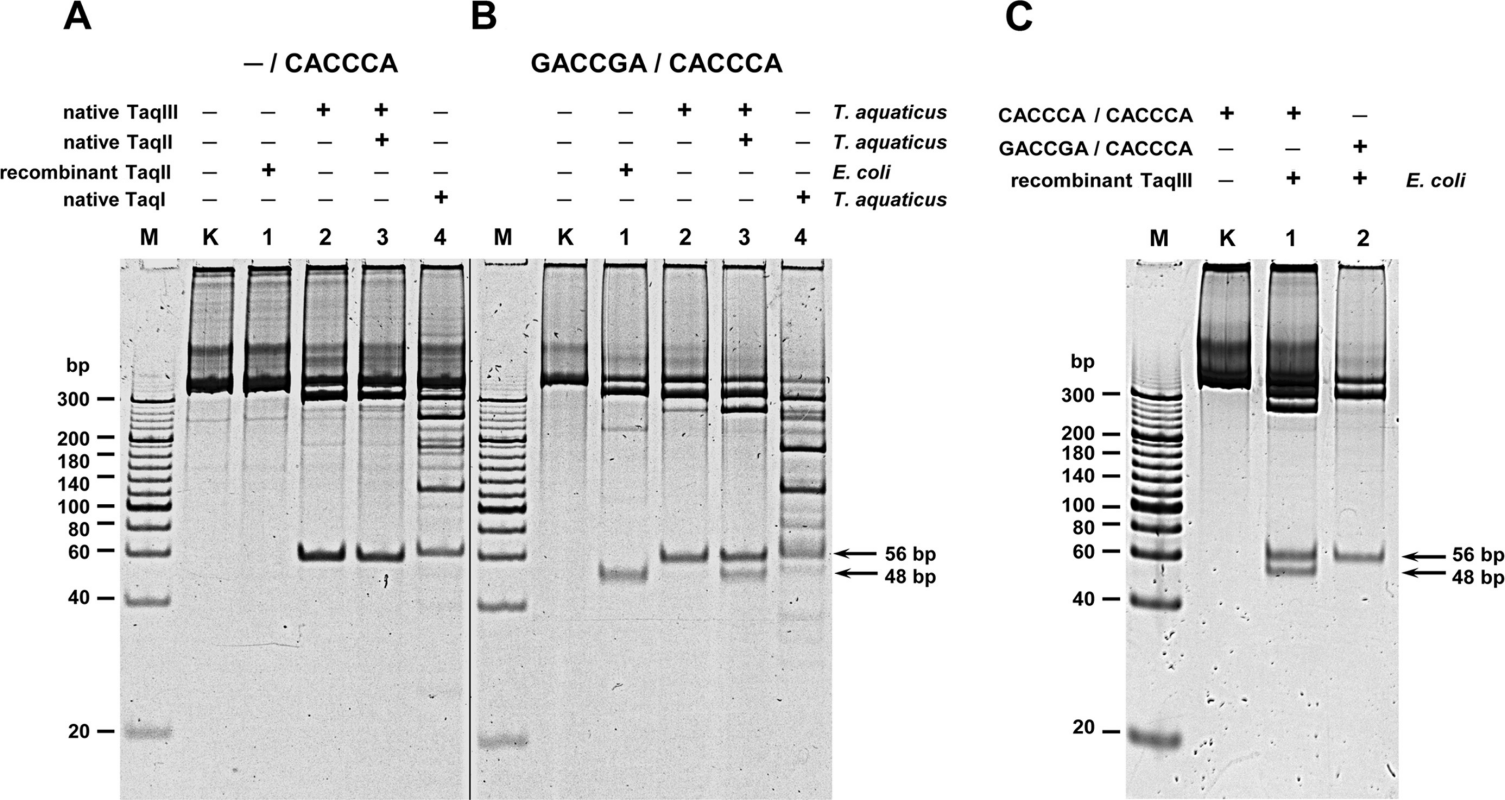
TaqIl and TaqIII cleavage patterns of PCR DNA substrates, containing 5′-CACCCA-3′ and 5′-GACCGA-3′ recognition sites. (**A**) Cleavage of the PCR (CACCCA) (←). Lane M, Sigma PCR 20-bp Low Ladder (selected bands marked); lane K, undigested PCR fragment; lanes 1–3, digested PCR fragment; lane 1, with recombinant TaqII; lane 2, with native TaqIII (fraction A); lane 3, with mixture of native TaqII and TaqIII (fraction B). (**B**) Cleavage of the PCR (GACCGA/CACCCA) (→←). Lanes M, 1–3, as in panel A. (**C**) Cleavage of the PCR substrates with recombinant TaqIII. Lane M, as in panel A; lane 1, digestion of the PCR(CACCCA/CACCCA) (→←); lane 2, digestion of the PCR(GACCGA/CACCCA) (→←).

To try to separate the two activities from a *T. aquaticus* lysate, we tested various chromatographic resins and binding conditions. Only Resource S cation exchange chromatography performed in 20 mM MES–Na buffer at pH 6.5 (slightly above the theoretically predicted pI of TaqII) resulted in partial separation of the two activities (Supplementary data), but it supported the concept of existence of a distinct TaqIII protein responsible for the 5′-CACCCA-3′ activity. The active fractions were further purified using Heparin–Agarose affinity chromatography and analysed by SDS-PAGE. In this manner, two enzyme preparations were obtained (Supplementary data). The first preparation (fraction A) contained proteins that did not bind to the Resource S column (concentrated flow-through and wash fraction). This preparation exhibited REase activity against 5′-CACCCA-3′ only (Figure 1, lane 1; Figure 2AB, lanes 2). The second preparation (fraction B) contained proteins that bound to Resource S resin and eluted at ∼ 550 mM NaCl. Fraction B exhibited REase activity against both 5′-GACCGA-3′ and 5′-CACCCA-3′ (Figure 1, lane 2; Figure 2AB, lanes 3). The presence of a 120 kDa protein was detected in both enzyme preparations (Figure 1, lanes 1,2). Interestingly, the second preparation contained two proteins, very similar in size, which could be separated only by SDS-PAGE on 6% gels (Figure 1, lane 2). Intriguingly, there is a difference in gel migration between native and recombinant enzymes. The predicted molecular weight (MW) of TaqII is 125.7 kDa, while the MW of recombinant TaqII, determined by SDS-PAGE and MALDI-TOF mass spectrometry, is 127.2 kDa (not shown). We hypothesize that the observed gel shifting may be caused by various types of post-translational modifications, occurring in *E. coli* and *T. aquaticus*. The gel shifting of homologous proteins is often discussed in the literature (35,36).

On the basis of presented results we assumed that fraction A could contain the presumed TaqIII only, while fraction B might be a mixture of TaqII and TaqIII.

### Mass spectrometry analysis confirmed the existence of TaqIII

In order to confirm the assumption presented in the previous section, we performed mass spectrometry analysis. The homogenous protein from fraction A was subjected to mass spectrometry LC–MS–MS/MS (37). The recombinant TaqII was used for control analysis (Supplementary data, Figure S2). The experimental peptide mass data were compared with predicted TaqII-derived peptide masses and with the NCBI non-redundant sequence database using the MASCOT search engine (http://www.matrixscience.com) (38). Both analysed proteins were identified as TaqII by database search (Supplementary data, Figure S2). However, investigating data obtained from the MS/MS analysis of the protein from fraction A we found 40 different peptides matching perfectly the TaqII aa sequence (Supplementary data, Figure S2) and 14 unique peptides, showing similarity to the TaqII aa sequence except for 1–3 replaced aa (Supplementary data, Table S1 and Figure S2A).

The MS–MS analysis confirmed that the REase from fraction A (presumed to be TaqIII) is a single protein, distinct from TaqII. The analysis revealed significant aa sequence similarity between the TaqII and TaqIII, especially within the PD-(D/E)XK nuclease domain, helical domain and RMF MTase domain of TaqIII (Supplementary data, Figure S2A). Nine of the fourteen peptides unique to TaqIII were located within a presumed target recognition domain (TRD) in the C-terminal part of the protein. This result indicates that the investigated proteins have variable aa sequence within this region. Such differences could explain the altered substrate specificity of TaqII and TaqIII.

### Western blot immunodetection showed significant similarity between TaqII and TaqIII

**Figure 3.**
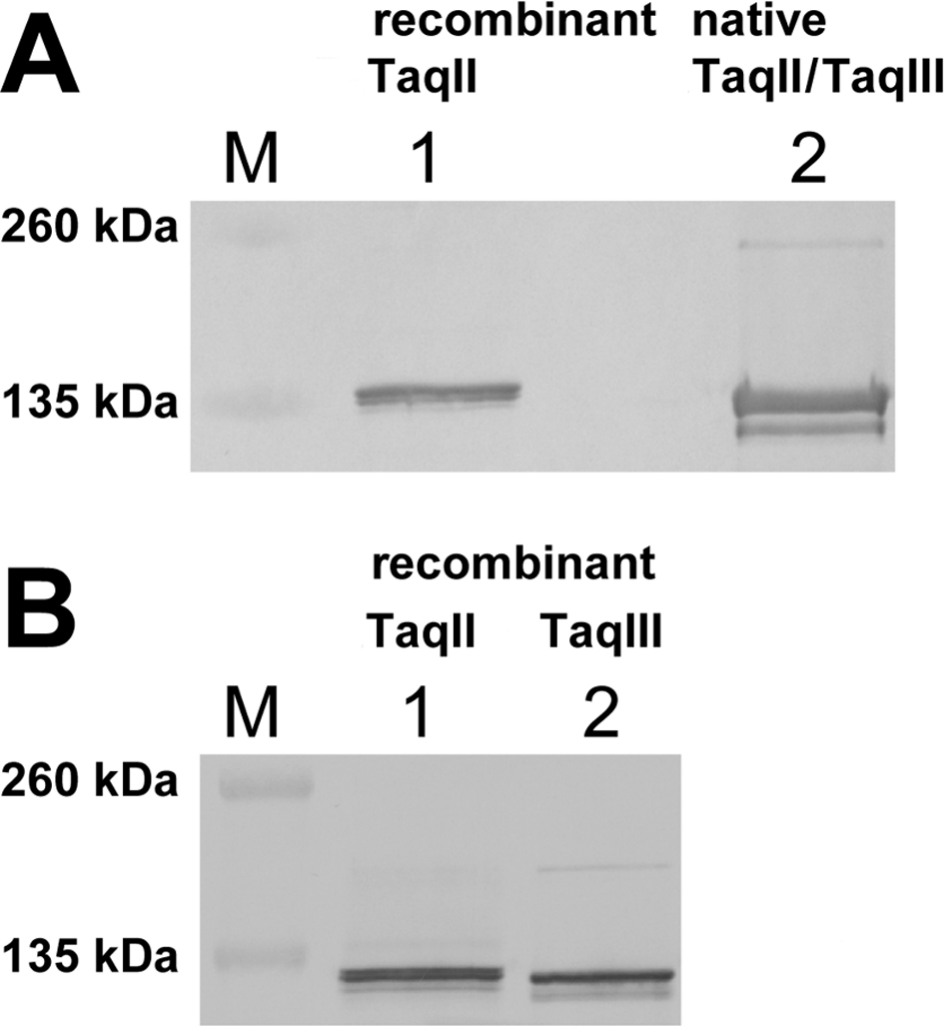
Immunodetection of native and recombinant TaqII and TaqIII proteins by anti-TaqII rabbit polyclonal antibody. (**A**) Immunodetection of native TaqII/TaqIII in the protein mixture. Lane M, protein marker (Thermo Fisher Scientific), bands marked: 260, 135 kDa; lane 1, control recombinant TaqII; lane 2, native TaqII/TaqIII protein mixture (fraction B, obtained from Resource S chromatography). (**B**) Immunodetection of recombinant protein variants, purified from *E. coli*. Lane M, protein marker (Thermo Fisher Scientific), bands marked: 260, 135 kDa; lane 1, recombinant TaqII; lane 2, recombinant TaqIII.

To address the question concerning the level of structural/sequence similarity between TaqII and the presumed TaqIII, western blot analysis was performed. Rabbit polyclonal anti-TaqII antibodies were used for immunodetection of native and recombinant TaqII and TaqIII protein variants. Interestingly, the anti-TaqII antibody could specifically detect all of the investigated proteins, whether of *T. aquaticus* (Figure 3A) or *E. coli* origin (Figure 3B). This confirmed significant similarity between the enzymes.

### Native TaqIII cleaves 5′-CACCCA-3′ only

The determination of the TaqIII DNA recognition sequence was performed using native protein, obtained from the fraction A (see Materials and Methods section). For that purpose three methods were used: (*i*) assessment of the digestion pattern on λ, pUC19 and pBR322 DNA, and custom devised substrates (10), containing both variants of the originally reported recognition sequence (20), (*ii*) direct DNA sequencing of TaqIII cleavage products and (*iii*) methylome analysis of *T. aquaticus* YT-1 genomic DNA.

Analysis of the native TaqIII cleavage pattern on several substrate DNA molecules confirmed that the recognition sequence is 5′-CACCCA-3′ (Figure 2; Supplementary data, Figures S3 and S4). However, all of these substrates were incompletely digested (Figure 2; Supplementary data, Figures S3 and S4), which is a characteristic feature of the *Thermus*-family enzymes. In contrast to recombinant TaqII, native TaqIII clearly does not cleave the 5′-GACCGA-3′ DNA sequence (Figure 2B, lane 2). Moreover, similarly to TaqII, TaqIII REase is stimulated by SAM and SIN (not shown) and is able to cleave linear molecules with a single DNA recognition sequence (Figure 2A, lane 2).

**Figure 4.**
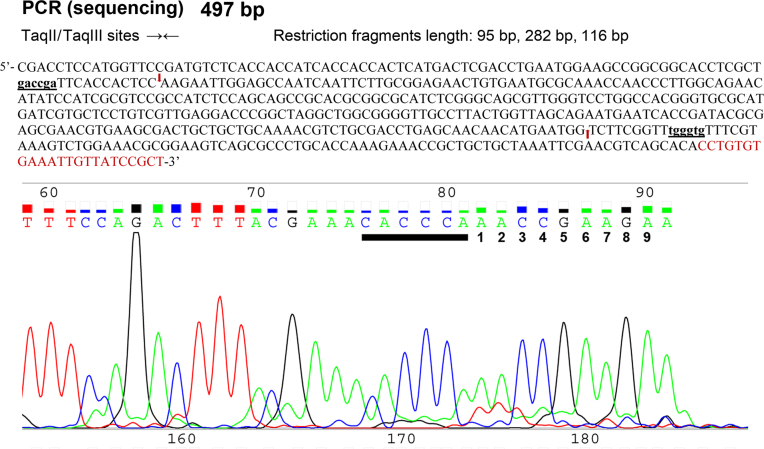
TaqIII DNA recognition site determination. DNA sequencing was performed using a 497 bp PCR fragment (10), cleaved with native TaqIII (fraction A). Putative recognition sequences of TaqII and TaqIII are in bold and underlined. Red arrows mark the cleavage sites. DNA sequence complementary to R22seq primer is in red. Run-off sequencing of the top strand of the native TaqIII-cleaved DNA fragment, adjacent to the 3′ DNA segment, flanking the 5′-CACCCA-3′ recognition site. The sequencing reverse R22seq primer governs polymerisation of the bottom strand with regard to 5′-CACCCA-3′, thus terminating at TaqIII-generated top-strand terminus. An additional base is added in a template-independent mode by the DNA polymerase included in the sequencing kit. Overlaying the uncut DNA region with both the top and bottom strand-sequencing data indicated a 2 bp gap. This was caused by 2 nt 3′-protruding termini generation by TaqIII cleavage. Thus, the top-strand cleavage point is located 11 nt downstream of the recognition site.

Direct confirmation of the recognition sequence came from run-off sequencing. A 497 bp PCR product, containing convergently oriented 5′-GACCGA-3′ and 5′-CACCCA-3′ sequences (10) was cleaved with native TaqIII and subjected to DNA sequencing (Figure 4). Sequencing from the 3′ end of the product clearly shows a cleavage point 9 nt downstream of the 5′-CACCCA-3′ site in the opposite strand (Figure 4).

**Figure 5.**
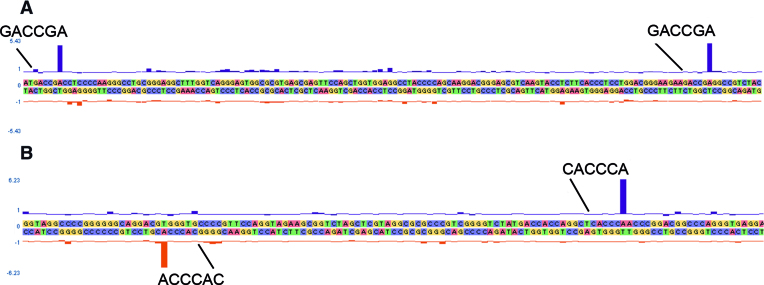
SMRT modification analysis of the TaqII and TaqIII system in *T. aquaticus* YT-1 DSM625. An IPD (Inter-Pulse-Duration) plot showing modification at a representative TaqII GACCGA^m6^ and TaqIII CACCCA^m6^ sequence motifs, within the *T. aquaticus* YT-1 genome sequence. (**A**) IPD ratios from a region including two TaqII recognition sites (GACCGA), both on the top strand. (**B**) IPD ratios from a region including two TaqIII recognition sites (CACCCA), one on each strand.

To identify TaqII and TaqIII DNA modifications and to confirm their specific DNA recognition motifs, genomic DNA from *T. aquaticus* YT-1 was SMRT sequenced. As expected, both modified 5′-GACCG(m6A)-3′ and 5′-CACCC(m6A)-3′ were found (Figure 5), with 100% of the TaqII and TaqIII motifs called as methylated at mean modification QV values of 403 (TaqII) and 438 (TaqIII). The modification of both motifs occurred on just one DNA strand, which is typical for Type IIL R–M systems.

The obtained results clearly demonstrate that native TaqIII recognizes the 5′-CACCCA-3′ DNA sequence for both cleavage and modification activities. This sequence had been originally attributed to TaqII as one of two distinct activities (20).

### *taqIIRM* and *taqIIIRM* genes are inter-plasmid paralogs

**Figure 6.**
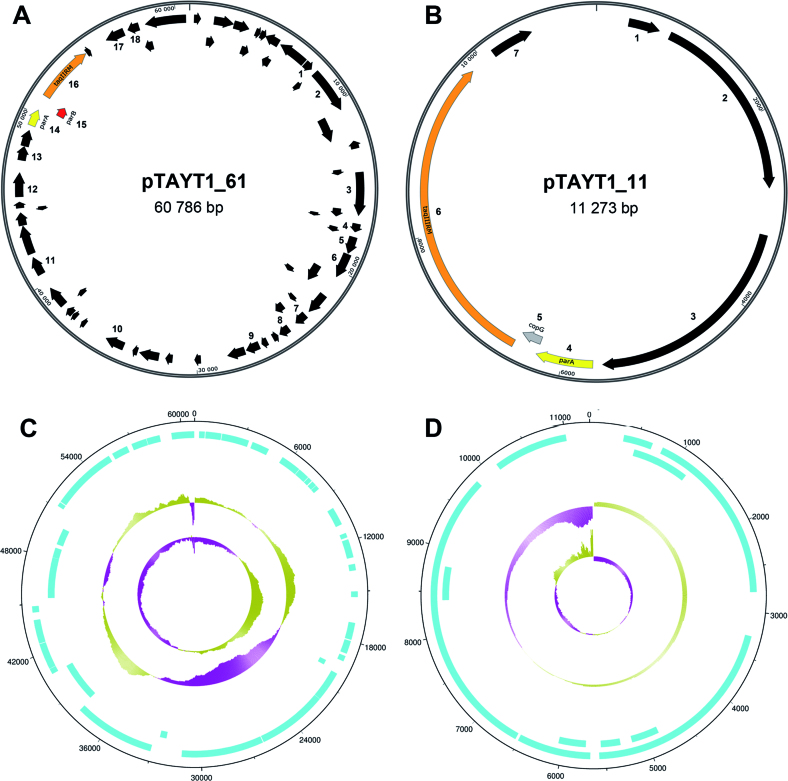
Localization of *taqIIRM* and *taqIIIRM* genes in the *T. aquaticus* YT-1 genome. (**A**) Circular representation of pTAYT1_61 plasmid. (**B**) Circular representation of pTAYT1_11 plasmid. ORFs are represented by black arrows. The *taqIIRM* and *taqIIIRM* genes are represented by orange arrows. The genes of the partitioning system are marked with yellow arrows (*parA* gene) and a red arrow (*parB* gene). The *copG* gene is marked with a grey arrow. Several ORF numbers and names are given for reference (see Tables S2 and S3 in Supplementary data). The schemes were created using SnapGene software. (**C**) Features of the *T. aquaticus* YT-1 plasmid pTAYT1_61. (**D**) Features of the *T. aquaticus* YT-1 plasmid pTAYT1_11. Tracks from outside to inside: CDS forward strand, CDS reverse strand, GC plot (%GC), GC skew ([GC]/[G+C]). For GC skew, the centre line indicates the average GC skew value for the genome. Green shading above the line denotes GC skew values greater than the genome average, whereas purple shading below the line denotes GC skew values less than the genome average. Prepared using DNA Plotter software (30).

Total genomic and plasmid DNA from *T. aquaticus* YT-1 was SMRT sequenced to generate complete, closed assemblies of the chromosome and all plasmids, in order to determine the *taqIIIRM* gene sequence. The *taqIIRM* and *taqIIIRM* ORFs were identified, 3318 and 3306 bp in length, respectively, and are highly similar in sequence (Supplementary data, Figure S5). Both ORFs start with an ATG codon but end with different STOP codons, TGA and TAG respectively. The two genes are encoded on two different plasmids: pTAYT1_11 (GenBank CP020571) and pTAYT1_61 (GenBank CP020572) (Figure 6; Supplementary data, Table S2 and S3). The G+C content of both genes is 66% and corresponds well to the G+C content of the respective plasmids pTAYT1_11 (65%) and pTAYT1_61 (69%) and the *T. aquaticus* YT-1 genome (68%). The *taqIIRM* and *taqIIIRM* genes and their flanking regions are devoid of TaqII and TaqIII recognition sites.

Plasmids pTAYT1_11 and pTAYT1_61 differ in size, at 11 and 61 kb, respectively. Both genes are in close proximity to a *parA* gene copy (Figure 6A and B). The *taqIIRM* gene is immediately preceded by *parB* and then *parA*, with the *parB* ORF overlapping the *taqIIRM* start codon. Based on putative promoter prediction, we speculate that *taqIIRM* might be a part of an operon that includes the *parAB* genes upstream. The *taqIIIRM* gene is immediately preceded by a *copG*-like gene and then *parA*, with the *parA* ORF overlapping the *copG* start codon; pTAYT1_11 lacks *parB*. We speculate that the putative *taqIIIRM* promoter is probably localized within the *copG* ORF and short intergenic region between *copG* ORF and *taqIIIRM* gene, as follows: 5′-aagttcTTGGCCactcaaagcgcacaaTAACCAGGGAcaggctc-3′, with –35 and –10 consensus sequences marked in capital letters (39,40). ParA and ParB proteins are responsible for predivisional partitioning of plasmid DNA molecules. The CopG protein belongs to the family of homologous plasmid repressors and is involved in regulating the plasmid copy number. The pTAYT1_61 DNA sequence is almost identical (sequence coverage 81%, 98% identity) to plasmid pTA69 (accession number CP010825.1) from *T. aquaticus* Y51MC23, whose genomic sequence has recently been published (41). There are two regions of dissimilarity in DNA sequence between pTAYT1_61 and pTA69. The *taqIIRM* gene comprises one of these regions and is replaced in pTA69 by an unrelated Type IIC/IIG R–M system annotated as ‘modification methylase BstVI’. The putative gene encodes a 1214 amino acid protein (possessing PD-(D/E)xK, NPPY and DPACGSG motifs and is proceeded by *parA/parB* genes. Moreover, the *T. aquaticus* Y51MC23 genome does not contain any gene encoding a *Thermus*-family R–M system, and no TaqII or TaqIII isoschizomers have been purified from other bacterial species so far (32).

### Sequence comparison of TaqII and TaqIII enzymes

**Figure 7.**
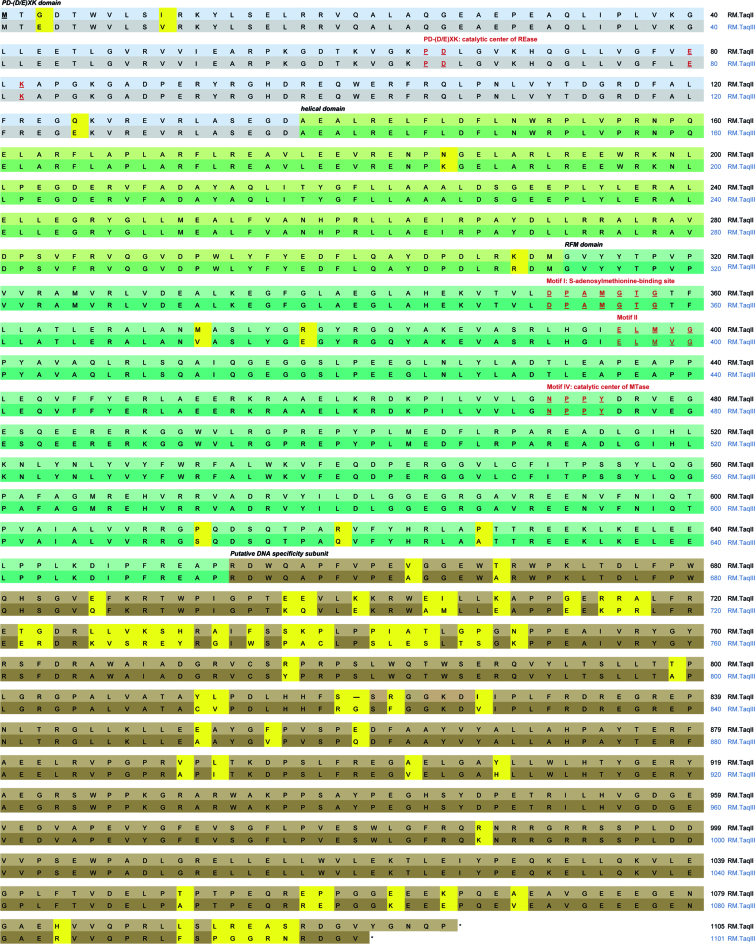
Comparison of the native TaqII and TaqIII aa sequences. The crucial aa of the catalytic centres are dark red, bold and underlined. The functional protein domains are marked as follows: REase domain in blue, helical domain in light green, MTase domain in dark green and the potential TRD region in brown. Differences in aa sequences between TaqII and TaqIII are marked in yellow.

Despite the striking similarity of TaqII and TaqIII in terms of physical properties (Table 1), the proteins could be separated by prolonged SDS-PAGE electrophoresis in 6% polyacrylamide gels (Figure 1). The aa sequences of TaqIII and TaqII are 93.4% identical (Figure 7; Supplementary data, Figure S5), which is the highest reported similarity of Type II REases that recognize different DNA sequences. Several putative TaqII/TaqIII homologs can be found in GenBank, providing a suitable target for the engineering of TaqII/TaqIII DNA specificity (Table 2).

**Table 1.** Comparison of physical and chemical parameters, computed for TaqII and TaqIII aa sequences

Parameters^1^	TaqII	TaqIII
No. of aa	1105	1101
Molecular weight	125.683	125.243
Theoretical pI	5.48	5.46
Total no. of negatively charged residues (Asp + Glu)	172	173
Total no. of positively charged residues (Arg + Lys)	147	148
No. of Cysteines	2	4
Instability index (II)^2^	41.68 (protein classified as unstable)	43.87 (protein classified as unstable)
Aliphatic index	88.53	87.70

^1^All parameters were computed using the ExPASy ProtParam tool (https://www.expasy.org).

^2^Based on their instability indices, both enzymes are classified as unstable proteins. The stability index is determined based on the protein primary structure alone. However, the stability of a protein *in vivo* is a net effect of the contributions made by several factors, such as: structure dependent features, the presence of disulphide bridges, ligand binding and protease recognition mechanisms (59).

**Table 2.** Putative IIC/IIG enzymes^a^ showing the highest sequence similarity to TaqIII

Organism	Protein	Length	Identities	*E*-value to TaqIII
*Thermus aquaticus* YT-1	TaqII	1105	1029	0.0
*Thermus* sp. GW	TspGWI	1097	668	0.0
*Anabaena* sp. 90	Putative Type IIC enzyme WP_015080118	1117	489	0.0
*Chromohalobacter israelensis*	Hypothetical protein WP_084076126	1088	493	0.0
*Nitrosospira briensis*	N-6 DNA Methylase SFN83300	1088	489	0.0
*Desulfobacca acetoxidans*	Hypothetical protein WP_013705953	1091	502	0.0
bacterium UASB270	DNA methyltransferase GAK56693	1107	476	0.0
*Oceanithermus profundus*	Protein of unknown function DUF450 WP_013449716	1102	536	0.0

^a^Identified by BLAST (blastp) search of the GeneBank non-redundant protein database.

The majority of the 71 aa differences between the two proteins occur within the putative TRD (Figure 7; Supplementary data, Table S4, Figure S5), consistent with the LC-MS-MS/MS analysis. All peptides detected for TaqIII by LC-MS-MS/MS (Supplementary data, Table S1, Figure S2) fit to the aa sequence derived from the *taqIIIRM* DNA sequence (not shown).

### Prediction of DNA recognition elements for TaqII and TaqIII

**Figure 8.**
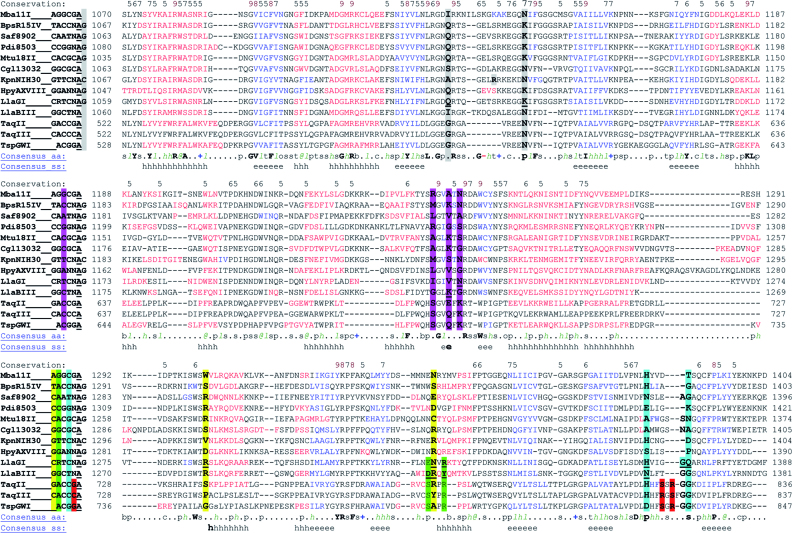
Amino acid sequence alignment of the TRD region for 10 LlaGI family Type ISP enzymes and 3 *Thermus*-family enzymes (TaqII, TaqIII and TspGWI) for which the recognition specificity is known. Highlight colors indicate aa positions that contact the highlighted base position within the aligned recognition motifs. Aa residues of LlaGI and LlaBIII that were observed to make direct base contacts in their crystal structures are underlined. Numbers indicate position within the protein; however note that in the text numbers are based on the LlaGI position for the Type ISP enzymes, and on TaqII for the *Thermus*-family enzymes. Consensus aa and Consensus ss (secondary structure) are as predicted by the promals alignment algorithm.

**Figure 9.**
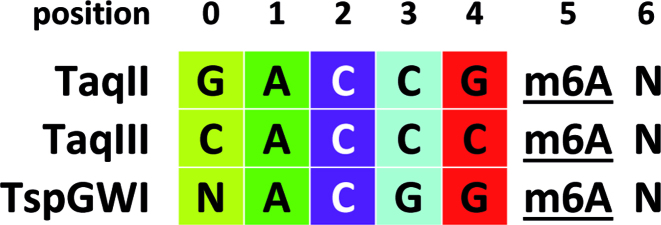
DNA recognition sequences in the TspGWI branch of the *Thermus*-family enzymes. Colors correspond to the highlighted base position within the aligned recognition motifs in the Figure 8. The fifth position is the adenine, which the enzymes methylate. The DNA strand that contains the methylated adenine is presented. N stands for no specificity.

Having proteins with highly similar aa sequences that yet recognize differing DNA sequence motifs, like TaqII and TaqIII, provides an opportunity to identify DNA recognition elements (positions and aa) through co-variation analysis (5). For the TaqII branch of the *Thermus*-family enzymes we currently have just three proteins for which the recognition motif is known. However the TRDs of TaqII and TaqIII have significant similarity to the TRDs of the Type ISP enzymes LlaGI and LlaBIII, for which crystal structures have recently been determined and residues making direct DNA contacts have been identified (42) (Supplementary data, Figure S6). We therefore aligned TaqII, TaqIII and TspGWI with the Type ISP enzymes described by Kulkarni, *et al* (42), and find that the TaqII and TaqIII proteins match well with the observed and co-variation-predicted DNA recognition elements in the Type ISP enzymes (Figure 8; Supplementary data, Figure S6). Note that in this discussion of recognition elements, we present the TaqII/TaqIII strand that contains the adenine which the enzymes methylate, and refer to the base positions as 0, 1, 2, 3, 4, 5 (and 6), where the fifth position is the methylated adenine, i.e.: TaqII 5′-GACCGA-3′ is 0 = G, 1 = A, 2 = C, 3 = C, 4 = G, 5 = (m6A), 6 = N (Figure 9).

At position 0, TaqII recognizes ‘G’, while TaqIII recognizes ‘C’, and TspGWI has no specificity, or ‘N’ (Figure 8 and 9). We predict that TaqII likely specifies ‘G’ through the Arg residue (R777) that aligns with the Arg residue in LlaBIII (R1327) that contacts the G in the top strand of the G:C base pair (bp) to specify ‘G’ at this position. TaqII also has a Ser residue (S737) at the aligned position where LlaGI uses an Arg residue (R1286) to specify ‘C’ recognition at position 0 (through contacts to the G in the bottom strand of this C:G bp). The pairing of Ser (737) and Arg (777) at these alignment positions is common in Type ISP TRDs and correlates to recognition of ‘G’ at position 0. TaqIII likely recognizes ‘C’ through these same alignment positions, with Tyr (Y777) likely contacting the ‘C’ base in the top strand, and Pro (P737) contacting the ‘G’ in the bottom strand, although this combination is uncommon. TspGWI, which does not specify a particular base at position 0, has small residues at these positions: Ser at 737 (S747) and Ala at 777 (A788), which likely allow any base to fit.

At position 1, TaqII, TaqIII and TspGWI all recognize ‘A’ (Figure 8 and 9), and all have the same residues, Ser and Arg, at the positions where LlaGI (R1329) and LlaBIII (D1326) make major-groove contacts to this base pair. LlaGI and LlaBIII make minor groove contacts to position 1 from residues within the MTase domain (S1056 in LlaGI; N1056 and N1058 in LlaBIII). In TaqII, TaqIII and TspGWI, the residues at these positions are larger (His at 1056 and Lys at 1058), which could potentially contribute to specificity for ‘A’ by excluding a G:C or C:G bp through steric clash with the guanine 2-amino group. Interestingly, LlaGI also contacts position 1 through the residue (N1327) at the alignment position where LlaBIII (R1327) specifies position 0 recognition. TaqII, TaqIII and TspGWI have different residues at this alignment position, yet all recognize ‘A’ at position 1, so it seems unlikely the *Thermus*-family enzyme residues corresponding to LlaGI position R1327 are making contacts to position 1, as is the case for LlaGI. Such subtle differences highlight the complexity and as yet poorly understood nuances of DNA recognition.

At position 2, the *Thermus*-family enzymes all specify ‘C’ (Figure 8 and 9), likely through contacts to the bottom strand ‘G" by their conserved Lys (K688) at the alignment position where LlaGI specifies ‘T’ through contacts by Asn (N1228) to the bottom strand ‘A’. Two nearby alignment positions, (S683 and E688) co-vary with the base recognized at position 2 in both the Type ISP and in the MmeI-family and thus may contribute to recognition as well.

For position 3 (Figure 8 and 9), the residue at the alignment position corresponding to LlaGI H1368 plays a critical role. His at this position co-varies with recognition of ‘C’, as in TaqII and TaqIII (H817), while a negative residue (Asp or Glu) co-varies with recognition of ‘G’, as in TspGWI (D828). The residues corresponding to LlaGI G1372 and Q1373 also contact this bp in the LlaGI co-crystal structure and thus may play a role in recognition for some enzymes, though for TaqII, TaqIII and TspGWI, these two alignment positions contain the same residues, Gly (G823) and Gly (G824), and so do not appear to be contributing directly to the difference in recognition.

For position 4 (Figure 8 and 9), LlaGI and LlaBIII are non-specific, and from their structures it was not obvious which aa positions might specify recognition in homologous enzymes. The *Thermus*-family enzymes have a small insertion of 2 (TaqII, TspGWI) or 3 (TaqIII) aa between the LlaGI position 3 contact positions H1368 and G1372, Q1373, suggesting this additional protein may contact the adjacent position 4 bp. Given the high degree of identity between the *Thermus*-family enzymes in this region, we predict that SXR or RXS at TaqII position S820 - R822 may specify recognition for a G:C (SXR) or a C:G (RxS) bp at position 4, wherein position 820 contacts the bottom strand base (S820 contacting ‘C' in TaqII and TspGWI; R820 contacting ‘G’ in TaqIII), and position 822 contacts the top strand base (R822 contacting ‘G’ in TaqII and TspGWI, S822 contacting ‘C’ in TaqIII). Whether the presence of an additional aa in TaqIII within this small putative loop may affect the positioning of the proposed RxS contacts is unknown, and further emphasizes the subtle nature of recognition within these enzymes.

Specificity at position 5 (Figure 8 and 9) is provided by the MTase domain, which flips the adenine into the methyltransferase catalytic pocket for recognition and eventual modification.

The *Thermus*-family enzymes do not have specificity at position 6 (Figures 8 and 9), which is consistent with the Type ISP findings, where non-specific enzymes have a small aa at the position corresponding to LlaGI Q1118 (G586 in TaqII) and an Asn at the position corresponding to LlaGI K1131 (N594 in TaqII). The *Thermus*-family enzymes also have a deletion of 7 aa within the LlaGI loop that contacts position 6, which likely contributes to their lack of specific DNA contacts.

The *Thermus*-family enzyme TRD domains are thus remarkably similar to those of the Type ISP enzymes, and the TaqII and TaqIII aa residues at the positions that make base-specific contacts in the LlaGI and LlaBIII structures are consistent with their observed recognition motifs. We plan to test this model of TaqII and TaqIII recognition elements through subsequent site-specific mutagenesis studies.

### Recombinant TaqIII protein

In order to finally establish that the identified *taqIIIRM* gene encodes the fully functional TaqIII protein, the gene was cloned into modified pRZ4737 vector, with gene expression driven by a λ P_R_ promoter that is inducible by a temperature shift to 42°C, as done for TaqII (16). A novel purification procedure was developed for purification of recombinant TaqII and TaqIII that uses PEI for selective acidic protein precipitation (25). The purified homogenous TaqIII protein was used for DNA cleavage assays (Figure 2C). Similarly to native protein, the recombinant TaqIII recognizes and cleaves the 5′-CACCCA-3′ DNA sequence only (Figure 2C).

## DISCUSSION

*T. aquaticus* YT-1 has two previously characterized R–M systems, TaqI (a Type II system recognizing 5′-TCGA-3′) and TaqII (a Type IIC system recognizing 5′-GACCGA-3′) (10,20). We report here the discovery of the third R–M system in *T. aquaticus* YT-1, TaqIII, a close homologue of TaqII recognizing the novel sequence 5′-CACCCA-3′. TaqII and TaqIII are members of the *Thermus* enzyme family (8), comprising closely related Type IIC R–M systems. TaqII and TaqIII enzymes represent an interesting example of naturally occurring sequence specificity evolution.

Because TaqII and TaqIII have highly similar protein sequences but different recognition motifs, they are excellent candidates for covariation analyses to identify specific recognition elements (5); however having just three characterized members form the TaqII branch of the *Thermus*-family severely limits covariation analysis. Interestingly, these enzymes share a conserved DNA recognition domain with the Type ISP enzymes, such as LlaGI and LlaBIII, even though these enzymes employ a completely different mechanism for DNA cutting. The excellent characterization of Type ISP DNA recognition carried out by the Szczelkun and Saikrishnan labs (42) can be applied to the TaqII family enzymes to help identify putative positions and aa that specify recognition, particularly those responsible for the differing specific recognition at two positions within the TaqII and TaqIII recognition motifs (Figure 8). We have used the analysis of recognition elements to predict recognition motifs for 6 uncharacterized *Thermus*-family enzymes (Supplementary data, Figures S6 and S7), which predictions can be readily tested. Based on our analysis of TaqII and TaqIII recognition elements, we anticipate it will prove possible to rationally alter their specificity at one or more positions.

The evolution of bacterial genomes is driven by HGT, mutation and genome rearrangement (43). From the other side, bacteria species are maintained by genetic isolation. R–M systems may facilitate bacterial genetic isolation by regulating the uptake of DNA from the environment (44). They also may establish favored patterns of genetic exchange between bacterial subpopulations (45).

It has been demonstrated that *Thermus* species acquire DNA through HGT (43). One of the major survival techniques of *Thermus* species is genome plasticity, which enables them to inhabit extreme temperature environments. Such unusual genome plasticity is achieved through natural transformation (46–48). Another characteristic feature of *Thermus* species is a remarkably high level of genome rearrangement (43,49). Frequently occurring (GC)_n_ repeats located upstream and downstream of the breakpoints of global genome rearrangements have been proposed to play a crucial role in facilitating homologous recombination (43).

*Thermus* genomes consist of chromosomes, megaplasmids and small plasmids (43). However, the number of plasmids per genome varies between bacterial strains (50). Our recent sequencing data revealed that *T. aquaticus* YT-1 genome contains a single chromosome of 2.08 Mb and six plasmids of sizes 8, 11, 12, 13, 61 and 71 kb (manuscript in preparation). The *taqIIRM* and *taqIIIRM* genes are localized on two separate plasmids: pTAYT1_61 and pTAYT1_11, respectively. The genes are probably paralogs, descended from an ancestral gene following a gene duplication event. Another scenario is that the genes are pseudoparalogs, acquired in two separate HGT events.

TaqII/TaqIII recognition sequences are absent in the *taqIIRM* and *taqIIIRM* genes but present in the *T. aquaticus* YT-1 genome. This is in agreement with the observation of Rusinov and co-workers that the sites Type I, IIC/IIG, IIM, III and IV R–M are usually not avoided (51).

Several independent studies have shown that R–M systems stabilize plasmids in cells by their selfish behaviour and have an impact on the host genome (52–55). Intriguingly, Oliveira and co-workers demonstrated that R–M systems are relatively rare in plasmids and other MGEs. In contrast to 69% of the investigated chromosomes, only 10.4% of the investigated plasmids encode R–M systems (56). Oliveira *et al*. linked the occurrence of R–M systems in plasmids with plasmid HGT via conjugation or via mobilization *in trans* using the conjugation machinery of another cell (56). However, one recent study revealed that the presence of R–M systems may limit the spread of naturally occurring plasmids between related bacterial species by mobilization (57). Moreover, it was shown that bacteria that possess plasmid-encoded R–M systems could efficiently release those plasmids into the environment, enabling their spread by natural transformation (57). It remains to be clarified whether and to what extent such mechanisms exist in naturally competent *Thermus* species.

There are several evolutionary advantages to the movement of genes from a bacterial chromosome to a plasmid. It has been suggested that *Thermus* strains move evolutionary modifying genes onto plasmids to boost their level of genetic plasticity (43). Plasmids are smaller and more quickly replicating DNA molecules, supporting microorganism propagation. They also exhibit higher rates of mutation, leading to the enrichment of gene variants within a population (58).

The most common plasmid-encoded R–M systems are compact Type IIC enzymes (1,56). Such systems account for more than a third of all Type II R–M systems and show a lower degree of purifying selection than orthodox Type II or Type I systems. Their compactness, weaker structural constraints, and simpler co-evolution of recognition sites can promote the diversification of the protein sequence and faster evolution of new specificities (56). This can help them to be more efficient in establishing in a new host (56). This is in agreement with our experience with *Thermus*-family enzymes. Most of the positive bacterial clones we obtained contained mutated R–M genes, resulting in protein variants with several aa substitutions. We speculate that IIL *Thermus*-family enzymes may have the ability to change specificity within the same bacterial host.

## ACCESSION NUMBERS

GenBank numbers: CP020571 and CP020572.

## Supplementary Material

Supplementary Data
