# Supplementary material for: The third restriction–modification system from Thermus aquaticus YT-1: solving the riddle of two TaqII specificities
Source: Nucleic Acids Res. 2017 Jul 12;45(15):9005–18. doi: 10.1093/nar/gkx599 (PMC5587805; doi:10.1093/nar/gkx599)
Supplement: Supplementary Data [file gkx599_Supp.pdf]

Supplementary Figures and Tables

The third restriction-modification system from *Thermus aquaticus* YT-1: solving the riddle of two TaqII specificities

Piotr M. Skowron[1,†], Brian P. Anton[2,†], Edyta Czajkowska[1], Joanna Zebrowska[1], Ewa Sulecka[1], Daria Krefft[1], Joanna Jezewska-Frackowiak[1], Olga Zolnierkiewicz[1], Malgorzata Witkowska[1], Richard D. Morgan[2], Geoffrey G. Wilson[2], Alexey Fomenkov[2], Richard J. Roberts[2] and Agnieszka Zylicz-Stachula[1,*]

[1] Department of Molecular Biotechnology, Faculty of Chemistry, University of Gdansk, Wita Stwosza 63, 80-308 Gdansk, Poland
[2] New England Biolabs, 240 County Road, Ipswich, MA 01938, USA

* To whom correspondence should be addressed. Tel: +48 58 523 52 40; Fax: +48 58 523 50 12; Email: a.zylicz-stachula@ug.edu.pl

†These authors contributed equally to the work. The authors wish it to be known that, in their opinion, the first 2 authors should be regarded as joint First Authors

The detailed purification procedure of native TaqII/TaqIII from *T. aquaticus* YT-1

T. aquaticus YT-1 colonies were washed from a Petri dish into 1 L of modified Lysogeny Broth (LB) [0.5% tryptose, 0.3% yeast extract, 0.2% NaCl, 0.001% dilution of 2.1 g/L stock Nitsch's trace elements, pH 7.2]. Cultures were grown for 12 h at 65°C with vigorous aeration. The inoculum was used to initiate 10 L bacterial culture in a biofermentor Bioflo 310 (New Brunswick Scientific, Edison, NJ, USA). The biofermentation parameters were: agitation (400-500 rpm), the dissolved oxygen 60%, pH 7.2 at 65°C. The bacteria were cultivated for 19 h at 65°C to stationary phase with pH control. At OD_{600} of 2.9, the culture was cooled to 4°C and the cells were centrifuged. The yield was 106 g from 10 L of bacterial culture.

The purification scheme for native TaqII/TaqIII from *T. aquaticus* YT-1 cells included the following stages:

1. *Lysis*. 100 g of bacterial cells was suspended in 5 volumes of buffer L [20 mM Tris-HCl, pH 8.0 at 25°C, 0.5 mM EDTA, 100 mM NaCl, 0.5% Triton X-100, 5 mM 2-mercaptoethanol (βMe), 0.1 mM PMSF, 1× protease inhibitor solution (the SigmaFAST™ Protease Inhibitor), 0.5 mg/ml chicken egg lysozyme]. After 30 min incubation at 4°C, the lysate was sonicated and centrifuged.
2. *DEAE-cellulose chromatography*. A hybrid batch/gravity flow procedure was used. The binding and initial washing steps were performed in a batch format to eliminate extraneous debris, and avoid column clogging. The clear lysate (580 ml) was mixed with 250 ml of DEAE-cellulose, previously equilibrated with buffer L1 [20 mM Tris-HCl, pH 8.0 at 25°C, 0.5 mM EDTA, 100 mM NaCl, 5 mM βMe, 0.1 mM PMSF]. The suspension was incubated for 1 h at 4°C with gentle agitation. Then the resin was

centrifuged (5 min, 700 × g), the supernatant was carefully removed and the resin was washed with 750 ml of buffer L1. The suspension was gently agitated for 20 min on a platform shaker at 4ºC and then centrifuged. After the initial wash, the resin was transferred to a column for additional washing and protein elution. Proteins were eluted with buffer D [20 mM Tris-HCl, pH 8.0 at 25ºC, 0.5 mM EDTA, 500 mM NaCl, 5 mM βMe, 0.1 mM PMSF].

3. *Polyethyleneimine (PEI) treatment*. PEI was gradually added to a concentration of 0.2%. After stirring for 30 min at 4ºC, the TaqII/TaqIII containing nucleic acid-acidic proteins-PEI complexes were pelleted by centrifugation (2000 × g, 5 min, 4ºC). In the next step, buffer with higher ionic strength was used to extract the TaqII/TaqIII enzymes out of the pellet. The pellet was dissolved in buffer P [20 mM Tris-HCl, pH 8.0 at 25ºC, 0.5 mM EDTA, 1 M NaCl, 5 mM βMe, 0.1 mM PMSF], the suspension was stirred for 3 h at 4ºC and then centrifuged and the supernatant was subjected to ammonium sulfate (AmS) fractionation.
4. *AmS fractionation*. For the purpose of protein precipitation, 50% saturation was applied using solid AmS and the suspension was stirred overnight, centrifuged (15,000 × g, 30 min, 4ºC), dissolved in buffer Q [20 mM Tris-HCl, pH 6.5 at 10ºC, 0.1 mM EDTA, 50 mM NaCl, 5 mM βMe, 0.1 mM PMSF] and dialysed against the same buffer.
5. *Resource Q chromatography*. Anion exchange was conducted in buffer Q using a 6 ml Resource™ Q ion exchange column and an automated Akta Pure chromatography system (GE Healtcare, Uppsala, Sweden), with a NaCl concentration gradient (50-400 mM in buffer Q). Fractions containing the TaqII/TaqIII proteins were collected and concentrated using a Vivaspin™ Turbo 15 centrifugal concentrator, MWCO 10 kDa (Sartorius, Goettingen, Germany).
6. *Resource S chromatography*. Cation exchange chromatography was conducted in buffer S [20 mM MES-Na, pH 6.5 at 10ºC, 10 mM $MgCl_2$, 5 mM βMe] using a 6 ml Resource™ S ion exchange column and an automated Akta Pure chromatography system (GE Healtcare, Uppsala, Sweden), with a NaCl concentration gradient (0-1 M in buffer S). Using this chromatographic step two enzyme fractions were obtained: (*i*) fraction A, containing TaqIII only, which did not bind to the cation exchanger and (*ii*) fraction B, containing a mixture of TaqII/TaqIII, which eluted at ~550 mM NaCl.
7. *Heparin-Agarose chromatography*. Both enzyme preparations were subjected to affinity chromatography on Heparin-Agarose. For that purpose, PD-10 desalting columns (GE Healtcare, Upsalla, Sweden) were used for rapid buffer exchange. After desalting, the protein preparations were loaded onto Heparin-Agarose containing gravity columns equilibrated with buffer H [20 mM Tris-HCl, pH 8.0 at 4ºC, 50 mM NaCl, 10 mM $MgCl_2$, 5 mM βMe, 5% glycerol]. The contaminating proteins were removed at 140 mM NaCl (in the buffer H). The TaqII and TaqIII proteins eluted at 300 mM NaCl (in buffer H). Finally, the purified proteins were concentrated using Vivaspin™ 500 centrifugal concentrator, MWCO 10 kDa (Sartorius, Goettingen, Germany).

The detailed purification procedure of recombinant TaqII and TaqIII

The purification scheme was used for both recombinant TaqII and TaqIII from *E. coli* cells and included the following stages:

1. *Lysis and heat treatment*. 40 g of bacterial cells was suspended in 10 volumes of buffer L_R [50 mM Tris-HCl, pH 7.9 at 25ºC, 0.1 mM EDTA, 50 mM NaCl, 0.5% Triton X-100, 5 mM 2-mercaptoethanol (βMe), 5% glycerol, 0.1 mM PMSF, 1× protease inhibitor solution (the SigmaFAST™ Protease Inhibitor), 0.5 mg/ml chicken egg lysozyme]. After 30 min incubation at 4ºC, the lysate was sonicated and centrifuged. The supernatant was incubated for 20 min at 70ºC and the denatured thermolabile *E. coli* proteins were removed by centrifugation.

2. *PEI treatment*. PEI was gradually added to the supernatant to a concentration of 0.2%. After stirring for 30 min at 4ºC, the nucleic acid-acidic proteins-PEI complexes were pelleted by centrifugation (2000 × g, 5 min). The pellet was resuspended in the buffer L_R containing 200 mM NaCl. The suspension was stirred for 10 min at 4ºC and centrifuged. This step effectively washed out most of the contaminating *E. coli* proteins. In the next step, the L_R buffer containing 600 mM NaCl was used to elute either TaqII or TaqIII recombinant protein from the pellet. The resulting suspension was then centrifuged and the obtained supernatant was subjected to AmS fractionation.
3. *AmS fractionation*. This stage was conducted at 4ºC in two phases. In the first step, 20% saturation was applied and contaminating proteins were removed. In the second stage, 40% saturation was applied, the suspension was stirred overnight, centrifuged, dissolved in buffer GF [20 mM Tris-HCl, pH 8.0 at 25ºC, 0.1 mM EDTA, 400 mM NaCl, 2.5 mM DTT] and dialysed against the same buffer.
4. *Size exclusion chromatography*. This step was performed using Akta Pure chromatography system and HiLoad 16/600 Superdex 200 PG column (GE Healthcare, Uppsala, Sweden). The column was equilibrated in buffer GF, protein preparations were concentrated using a Vivaspin™ Turbo 15 centrifugal concentrator and subjected to molecular sieving. The purified protein preparations were concentrated, dialysed against storage buffer [20 mM Tris-HCl, pH 8.3 at 25ºC, 200 mM KCl, 25 mM AmS, 0.1 mM EDTA, 0.05% Triton X-100, 0.5 mM DTT, 50% glycerol] and stored at -20°C.

The detailed Western blotting and immunodetection procedure

Purified proteins were separated by SDS-PAGE in 10% gels and electroblotted onto a PVDF membrane (1), using the semi-dry Trans-Blot Turbo Transfer System and ready-to-use preassembled Trans-Blot Turbo Mini PVDF Transfer Pack (Bio-Rad) at 1.3 mA, 25 V for 30 min in Towbin buffer [25 mM Tris, 192 mM Glycine, pH 8.6 at 25ºC], with 20% methanol. The membrane was blocked at 4ºC for 1 h in TBS-T buffer [50 mM Tris-HCl, 150 mM NaCl, 0.1% Tween 20, pH 7.5 at 25ºC] with 3% skim milk. The membrane was then probed with rabbit anti-TaqII antibodies (courtesy of Dr D. Nidzworski, University of Gdansk, Poland) diluted 1:1000 in TBS-T buffer with 3% skim milk for 1 h at 37ºC. After three washes with TBS-T, the membrane was incubated for 1 h with goat anti-rabbit secondary antibody conjugated with alkaline phosphatase (AP, 1:10000) (Santa Cruz Biotechnology, courtesy of Dr D. Nidzworski, University of Gdansk, Poland) for 1 h 37ºC. The membrane was washed three times with TBS-T buffer and a specific protein was visualized by adding BCIP/NBT solution.

LC-MS-MS/MS analysis

LC-MS-MS/MS analysis (liquid chromatography coupled to tandem mass spectrometry) were performed at a Mass Spectrometry Laboratory (IBB PAS, Warsaw). Gel slices containing TaqII or TaqIII protein were subjected to a standard ‘in-gel digestion’ procedure. Any protein disulphide bonds were reduced with 100 mM DTT (30 min at 56ºC), alkylated with iodoacetamide (45 min; in a darkroom; room temperature) and digested overnight with trypsin (sequencing Grade Modified Trypsin, Promega). The resulting peptides were eluted from the gel with 0.1% trifluoroacetic acid (TFA) and 2% acetonitrile (ACN) and measured by LC/MS. HPLC separation parameters: precolumn: RP-18 (nanoACQUITY Symmetry R ® C18,Waters), 0.1% TFA as a mobile phase. Nano-HPLC: RP-18 column (nanoACQUITY BEH C18, Waters), flow rate 250 nl/min, gradient: 0-

35% B in 70 min, solvent A: 0.05% formic acid in water, solvent B: 0.05% formic acid in ACN. The column outlet was directly coupled to the ion source of the spectrometer working in the regime of data dependent MS to MS/MS switch (Orbitrap Velos mass spectrometer-Thermo Electron Corp.). The raw data were processed using Mascot Distiller followed by Mascot Search (Matrix Science, UK) against the predicted TaqII derived reference peptide masses. The search parameters for the precursor and product ions mass tolerance were 20 ppm and 0.1 Da, respectively; trypsin specificity: one missed cleavage sites allowed; fixed modification of cysteine by carbamidomethylation and variable modification and oxidation of methionine. Peptides with a Mascot Score exceeding the 5% False Positive Rate threshold and with a Mascot Score exceeding 30 were considered to be positively identified.

Cloning of the *taqIIIRM* gene

Nested PCR.

In the first step, two primers complementary to the pTAYT1_11 plasmid were used to specifically amplify a 3506 bp fragment containing *taqIIIRM*: F_taqIII_nested 5’-GGCTCTGAGGAAGTTCTTGG-3’ and R_taqIII_nested 5’-CTCTCCGCATAAACTCCGCA-3’. The 50 µl PCR reactions contained: 1× Q5 reaction buffer, 0.2 mM of each dNTP, 0.5 µM of each primer, 140 ng of template DNA (total plasmid DNA isolated from *T. aquaticus* YT-1), 3% DMSO and 1 unit of Q5® high fidelity DNA polymerase. The PCR cycling profile was as follows: 98°C for 30 sec, 98°C for 5 sec, 59°C for 10 sec, and 72°C for 1 min (for 25 cycles); 72°C for 2 min. The PCR product was subjected to agarose electrophoresis, gel isolated, and used as the template in a second round of nested PCR. The second round primers, complementary to the *taqIIIRM* gene, introduced BspHI and SalI sites (bold underlined): F_*taqIIIRM*_BspHI 5’-CCCC**<u>TCATGA</u>**CGGAAGACACTTGGGTCCTCA-3’ and R_*taqIIIRM*_SalI 5’-CCCC**<u>GTCGAC</u>**TTAACTAGACCCCGTCCCTGTTTC-3’. F_*taqIIIRM*_BspHI is also complementary to the *taqIIRM* gene. The 50 µl PCR reactions contained: 1× Q5 reaction buffer, 0.2 mM dNTPs, 0.5 µM of each primer, 47 ng of template DNA (the 3506 bp PCR fragment), 1× Q5 High GC Enhancer and 1 unit of Q5® high fidelity DNA polymerase. The PCR cycling profile was as follows: 98°C for 30 sec, 98°C for 5 sec, 57°C for 10 sec, and 72°C for 1 min (for 25 cycles); 72°C for 2 min. The resulting 3326 bp PCR fragment was subjected to agarose electrophoresis, gel isolated and used for *taqIIIRM* cloning. Restriction mapping of the product with BsaI and XbaI was performed to confirm the purity of the amplified t*aqIIIRM* gene. The presence of the *taqIIRM* gene was not detected.

The recombinant t*aqIIIRM* gene expression procedure

Positive bacterial clones were subjected to gene expression. *E. coli* BL21 Star™ (DE3) were electroporated with pRZ-*taqIIIRM*, which expresses *taqIIIRM*. Cells were grown in a 10 ml 24 deep well plate in 4 ml TB media supplemented with CM and maltose at 28°C with vigorous aeration. The temperature of 28°C was used to minimize residual TaqIII REase activity, decreasing its toxicity for a bacterial host. The P_R promoter was induced by a temperature shift to 42°C at OD_{600} of 0.8. Growth was continued for 4.5 h at 42°C. Bacterial pellets were subjected to SDS/PAGE. The gels were analysed for the appearance of the expected band size of ~120 kDa and for TaqIII REase activity in crude lysates, pre-incubated for 20 min at 70°C. Bacterial clones efficiently expressing *taqIIIRM* gene were selected for a large-scale bacterial culture.

DNA cleavage assays

All TaqIII cleavage reactions were carried out in [Tris-HCl, pH 7.2 at 25ºC, 10 mM $MgCl_2$, 1 mM DTT, BSA 100 µg/ml] at 70ºC. The protein-to-DNA recognition site molar ratio differs depending on the experiment. The amount of the substrate DNA was 0.3 µg and the reaction volume was 50 µl. After incubation at 70ºC, the reactions were quenched with phenol/chloroform, and DNA was ethanol-precipitated, centrifuged and dissolved in 10 mM Tris-HCl, pH 8.0 at 25ºC. The products were analyzed by electrophoresis in either 1.2% agarose or 15% polyacrylamide gels. Gels were prepared in 1× Tris-Borate-EDTA (TBE) buffer (1). Agarose gels were stained with ethidium bromide. The polyacrylamide gels were stained with Sybr Gold.

Determination of TaqIII recognition sequence and cleavage site

DNA fragments from TaqIII digestion of a 497 bp substrate (2) were subjected to PAGE and isolated using electroelution, 1× phenol/chloroform, 2× chloroform extractions and ethanol precipitation. The purified restriction fragments were sequenced using the ABI Prism 310 automated sequencer with ABI Prism BigDye Terminator Cycle Sequencing Ready Reaction Kit (Perkin Elmer Applied Biosystems, Foster City, CA, USA). The run-off sequencing reactions were performed with primers: F18seq-390 5'-CGACCTCCATGGTTCCGA-3' and R22seq-390 5'-AGCGGATAACAATTTCACACAGG-3' (2).

REFERENCES

1. Green,M.R. and Sambrook,J. (2012). Molecular Cloning: A Laboratory Manual, 4th ed. Cold Spring Harbor Laboratory Press, CSH, NY.

2. Żylicz-Stachula,A., Żołnierkiewicz,O., Śliwińska,K., Jeżewska-Frąckowiak,J. and Skowron,P.M. (2011) Bifunctional TaqII restriction endonuclease: redefining the prototype DNA recognition site and establishing the Fidelity Index for partial cleaving. *BMC Biochem.*, **12**:62.

SUPPLEMENTARY TABLES AND FIGURES LEGENDS

A

PCR (-/CACCCA) 390 bp **TaqIII site ←** **Restriction fragments length:** 332 bp, 56 bp

5'- CTCGACCTGAATGGAAGCCGGCGGCACCTCGCTAACGGATTCACCACTCCAAGAATTGGAGCCAATCAATTCTTGCGG
AGAACTGTGAATGCGCAAACCAACCCTTGGCAGAACATATCCATCGCGTCCGCCATCTCCAGCAGCCGCACGCGGCGC
ATCTCGGGCAGCGTTGGGTCCTGGCCACGGGTGCGCATGATCGTGCTCCTGTCGTTGAGGACCCGGCTAGGCTGGCGGG
GTTGCCTTACTGGTTAGCAGAATGAATCACCGATACGCGAGCGAACGTGAAGCGACTGCTGCTGCAAAACGTCTGCGA
CCTGAGCAACAACATGAATGGTCTTCGGTT**<u>tgggtg</u>**TTTCGTAAAGTCTGGAAACGCGGAAGTCAGCGCCCTGCACC-3'
↑9 8 7 6 5 4 3 2 1

B

PCR (CACCCA/CACCCA) 390 bp **TaqIII sites →←** **Restriction fragments length:** 48 bp, 282 bp, 56 bp

1 2 3 4 5 6 7 8 9 10 11↓
5'- CTCGACCTGAATGGAAGCCGGCGGCACCTCGCT**<u>caccca</u>**TTCACCACTCCAAGAATTGGAGCCAATCAATTCTTGCGGAG
AACTGTGAATGCGCAAACCAACCCTTGGCAGAACATATCCATCGCGTCCGCCATCTCCAGCAGCCGCACGCGGCGCAT
CTCGGGCAGCGTTGGGTCCTGGCCACGGGTGCGCATGATCGTGCTCCTGTCGTTGAGGACCCGGCTAGGCTGGCGGGGT
TGCCTTACTGGTTAGCAGAATGAATCACCGATACGCGAGCGAACGTGAAGCGACTGCTGCTGCAAAACGTCTGCGACC
TGAGCAACAACATGAATGGTCTTCGGTT**<u>tgggtg</u>**TTTCGTAAAGTCTGGAAACGCGGAAGTCAGCGCCCTGCACC-3'
↑9 8 7 6 5 4 3 2 1

C

PCR (GACCGA/CACCCA) 390 bp **TaqII site →← TaqIII site** **Restriction fragments length:** 48 bp, 282 bp, 56 bp

1 2 3 4 5 6 7 8 9 10 11↓
5'- CTCGACCTGAATGGAAGCCGGCGGCACCTCGCT**<u>gaccga</u>**TTCACCACTCCAAGAATTGGAGCCAATCAATTCTTGCGGAG
AACTGTGAATGCGCAAACCAACCCTTGGCAGAACATATCCATCGCGTCCGCCATCTCCAGCAGCCGCACGCGGCGCAT
CTCGGGCAGCGTTGGGTCCTGGCCACGGGTGCGCATGATCGTGCTCCTGTCGTTGAGGACCCGGCTAGGCTGGCGGGGT
TGCCTTACTGGTTAGCAGAATGAATCACCGATACGCGAGCGAACGTGAAGCGACTGCTGCTGCAAAACGTCTGCGACC
TGAGCAACAACATGAATGGTCTTCGGTT**<u>tgggtg</u>**TTTCGTAAAGTCTGGAAACGCGGAAGTCAGCGCCCTGCACC-3'
↑9 8 7 6 5 4 3 2 1

Figure S1. PCR fragment DNA substrates. The putative recognition sequences of TaqII and TaqIII are in bold and underlined. Arrows mark the points of cleavage. (A) PCR DNA fragment with a single 5'-CACCCA-3' site (←). (B) PCR DNA fragment with two convergent 5'-CACCCA-3' sites (→←). (C) PCR DNA fragment with both 5'-GACCGA-3' and 5'-CACCCA-3' sites (→←). PCR fragments were obtained, using suitable combinations of the following primers (the introduced TaqII/TaqIII recognition sequence is in bold and underlined):
5'-CTCGACCTGAATGGAAGCCG-3',
5'-GGTGCAGGGCGCTGACTTCC-3',
5'-CTCGACCTGAATGGAAGCCGGCGGCACCTCGCT**<u>GACCGA</u>**TTCACCACT-3',
5'-CTCGACCTGAATGGAAGCCGGCGGCACCTCGCT**<u>CACCCA</u>**TTCACCACT-3',
5'-GGTGCAGGGCGCTGACTTCCGCGTTTCCAGACTTTACGAAA**<u>CACCCA</u>**AACCGAAGA-3'.

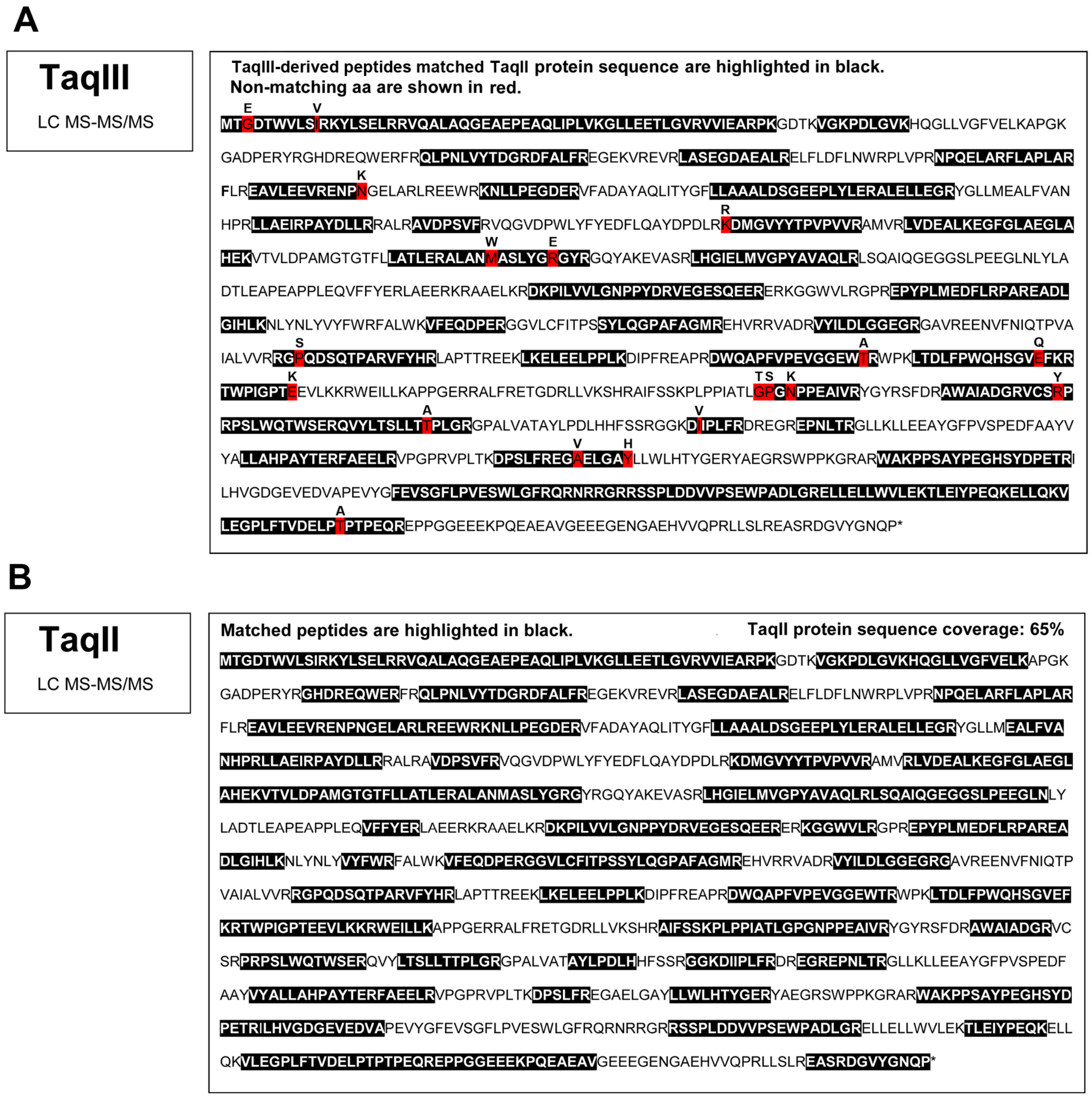

Figure S2. TaqII amino acid sequence coverage by LC-MS-MS/MS spectrometry. (A) Amino acid sequence of TaqII. TaqIII peptide sequences identified from MS-MS data (Table S1) are highlighted in black. Non-matching amino acids are highlighted in red. (B) Amino acid sequence of TaqII. Control TaqII peptide sequences identified from MS-MS data are highlighted in black.

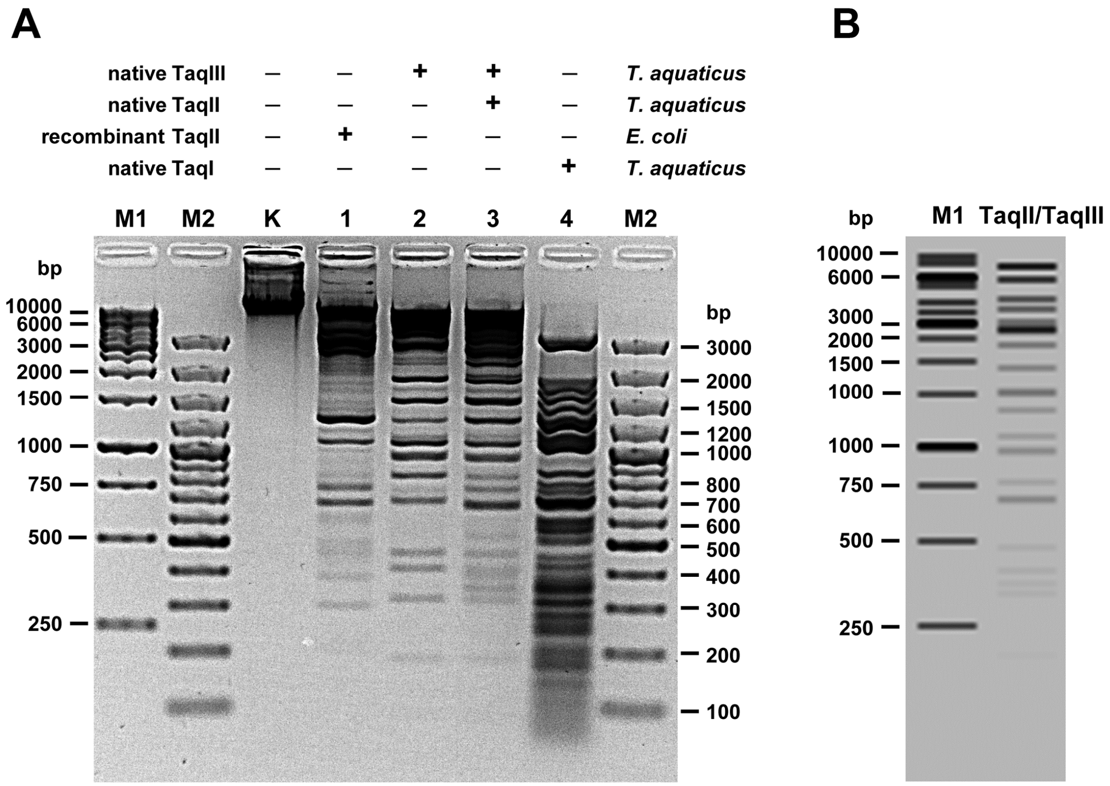

Figure S3. TaqII/TaqIII cleavage patterns of bacteriophage lambda DNA. (A) Lane M1, GeneRuler^{TM} 1 kb DNA Ladder (Thermo Fisher Scientific/Fermentas), selected bands marked; lane M2, GeneRuler^{TM} 100 bp DNA Ladder (Thermo Fisher Scientific/Fermentas), selected bands marked; lane K, untreated λ DNA; lane 1, recombinant TaqII-cut λ DNA; lane 2, native TaqIII-cut λ DNA (fraction A, obtained from Resource S chromatography); lane 3, native TaqII/native TaqIII-cut λ DNA (fraction B, obtained from Resource S chromatography); lane 4, TaqI-cut λ DNA. Reaction products were resolved by 1.2% agarose gel electrophoresis in 1× TBE buffer and stained with ethidium bromide. (B) The predicted TaqII/TaqIII cleavage pattern of lambda DNA (SnapGene).

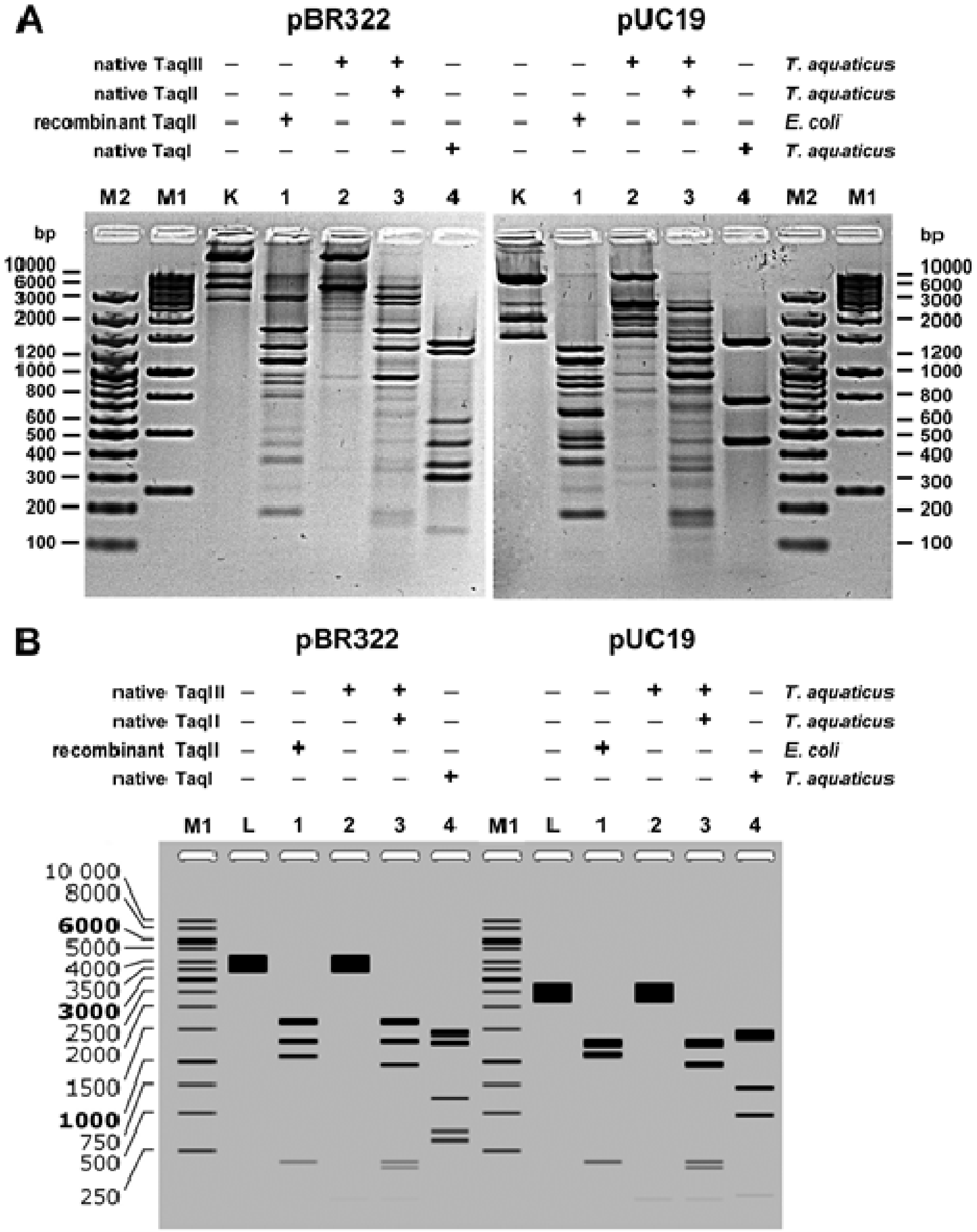

Figure S4. TaqII/TaqIII cleavage patterns of reference plasmid DNA substrates. (A) Cleavage pattern of pBR322 and pUC19 plasmid DNA. Lane M1, GeneRuler™ 1 kb DNA Ladder (Thermo Fisher Scientific/Fermentas), selected bands marked; lane M2, GeneRuler™ 100 bp DNA Ladder (Thermo Fisher Scientific/Fermentas), selected bands marked; lane K, untreated plasmid DNA; lane 1, recombinant TaqII-cut plasmid DNA; lane 2, native TaqIII-cut plasmid DNA (fraction A, obtained from Resource S chromatography); lane 3, native TaqII/native TaqIII-cut plasmid DNA (fraction B, obtained from Resource S chromatography); lane 4, TaqI-cut plasmid DNA. Reaction products were resolved on 1.2% agarose gel electrophoresis in 1× TBE buffer and stained with ethidium bromide. (B) The predicted cleavage patterns of pBR322 and pUC19 plasmid DNA (SnapGene). Lane L, linear form of plasmid DNA; lanes 1-4, as in A.

PD-(D/E)XK domain

ATG ACG GGA GAC ACT TGG GTC CTC AGT ATA AGG AAA TAC CTA AGC GAG CTT CGC CGG GTT CAG GCC CTA GCC CAG GGC GAG GCC GAG CCC GAG GCC CAG CTC ATC CCG TTG GTG AAG GGG **120** *wttaqIIRM*

ATG ACG GAA GAC ACT TGG GTC CTC AGT GTA AGG AAA TAC CTA AGT GAG CTT CGC CGG GTT CAG GCC CTA GCC CAG GGC GAG GCC GAG CCC GAG GCC CAG CTC ATC CCG TTG GTG AAG GGG 120 *wttaqIIIRM*

M T G D T W V L S I R K Y L S E L R R V Q A L A Q G E A E P E A Q L I P L V K G 40 RM.TaqII

M T E D T W V L S V R K Y L S E L R R V Q A L A Q G E A E P E A Q L I P L V K G 40 RM.TaqIII

PD-(D/E)XK: catalytic center of REase

CTT TTG GAG GAA ACC CTG GGG GTG CGG GTG GTC ATA GAG GCC CGT CCC AAA GGG GAT ACC AAA GTG GGC AAG CCG GAC CTA GGG GTG AAG CAC CAG GGG CTC CTG GTG GGC TTC GTG GAG **240** *wttaqIIRM*

CTT TTG GAG GAA ACC CTG GGG GTG CGG GTG GTC ATT GAG GCC CGC CCC AAA GGG GAT ACC AAA GTG GGC AAG CCG GAC CTA GGG GTG AAG CAC CAG GGG CTC CTG GTG GGC TTC TTG GAA 240 *wttaqIIIRM*

L L E E T L G V R V V I E A R P K G D T K V G K **P** **D** L G V K H Q G L L V G F V **E** 80 RM.TaqII

L L E E T L G V R V V I E A R P K G D T K V G K **P** **D** L G V K H Q G L L V G F L **E** 80 RM.TaqIII

CTC AAG GCT CCG GGC AAG GGG GCC GAC CCC GAA AGG TAC AGG GGC CAC GAC CGG GAG CAG TGG GAG AGG TTC CGC CAG CTA CCC AAT CTG GTC TAC ACG GAC GGG CGG GAC TTC GCC CTC **360** *wttaqIIRM*

CTC AAG GCC CCG GGC AAG GGG GCC GAC CCC GAA AGG TAC AGG GGC CAC GAC CGG GAG CAG TGG GAG AGG TTC CGC CAG CTA CCC AAT CTG GTC TAC ACG GAC GGG CGG GAC TTC GCC CTC 360 *wttaqIIIRM*

L **K** A P G K G A D P E R Y R G H D R E Q W E R F R Q L P N L V Y T D G R D F A L 120 RM.TaqII

L **K** A P G K G A D P E R Y R G H D R E Q W E R F R Q L P N L V Y T D G R D F A L 120 RM.TaqIII

helical domain

TTC CGC GAG GGG CAG AAG GTC CGA GAG GTG CGG CTA GCC TCG GAG GGC GAC GCC GAA GCT CTG CGG GAG CTC TTC CTG GAC TTC CTC AAC TGG AGG CCC CTG GTC CCT AGG AAC CCC CAG **480** *wttaqIIRM*

TTC CGC GAG GGG GAG AAG GTC CGA GAG GTG CGA CTG GCC TCG GAA GGC GAC GCC GAA GCT CTG CGG GAG CTC TTC CTG GAC TTC CTC AAC TGG AGG CCC CTG GTC CCC AGG AAC CCC CAG 480 *wttaqIIIRM*

F R E G Q K V R E V R L A S E G D A E A L R E L F L D F L N W R P L V P R N P Q 160 RM.TaqII

F R E G E K V R E V R L A S E G D A E A L R E L F L D F L N W R P L V P R N P Q 160 RM.TaqIII

GAG CTG GCC CGC TTC CTC GCC CCC CTG GCC CGC TTC CTG CGG GAG GCG GTG CTG GAG GAG GTG AGG GAG AAC CCA AAC GGA GAA CTG GCG CGT CTT CGC GAA GAG TGG CGC AAA AAC CTC **600** *wttaqIIRM*

GAG CTG GCC CGC TTC CTC GCC CCC CTG GCC CGC TTC CTG CGG GAG GCG GTG CTG GAG GAG GTG AGG GAA AAC CCA AAG GGG GAA CTG GCG CGT CTT CGG GAA GAG TGG CGC AAA AAC CTC 600 *wttaqIIIRM*

E L A R F L A P L A R F L R E A V L E E V R E N P N G E L A R L R E E W R K N L 200 RM.TaqII

E L A R F L A P L A R F L R E A V L E E V R E N P K G E L A R L R E E W R K N L 200 RM.TaqIII

CTG CCC GAG GGG GAT GAG AGG GTC TTC GCC GAC GCC TAC GCC CAG CTC ATC ACC TAC GGC TTC CTC CTG GCC GCG GCC CTG GAC AGC GGG GAG GAG CCC CTC TAC CTG GAG CGG GCC CTG **720** *wttaqIIRM*

CTG CCC GAG GGG GAT GAG AGG GTC TTC GCC GAC GCC TAC GCC CAG CTC ATC ACC TAC GGC TTC CTC CTG GCC GCG GCC CTG GAC AGC GGG GAG GAG CCC CTC TAC CTG GAG CGG GCT CTG 720 *wttaqIIIRM*

L P E G D E R V F A D A Y A Q L I T Y G F L L A A A L D S G E E P L Y L E R A L 240 RM.TaqII

L P E G D E R V F A D A Y A Q L I T Y G F L L A A A L D S G E E P L Y L E R A L 240 RM.TaqIII

GAG CTC CTG GAG GGG CGG TAC GGC CTC CTC ATG GAG GCC CTC TTC GTG GCC AAC CAC CCC CGG CTC CTG GCG GAG ATC CGC CCT GCC TAC GAC CTC CTG CGG CGG GCC CTC CGG GCG GTG **840** *wttaqIIRM*

GAG CTC CTG GAG GGG CGG TAC GGC CTC CTC ATG GAG GCC CTC TTC GTG GCC AAC CAC CCC CGG CTC CTG GCG GAG ATC CGC CCC GCC TAC GAC CTC CTG CGG CGG GCC CTC CGG GCG GTG 840 *wttaqIIIRM*

E L L E G R Y G L L M E A L F V A N H P R L L A E I R P A Y D L L R R A L R A V 280 RM.TaqII

E L L E G R Y G L L M E A L F V A N H P R L L A E I R P A Y D L L R R A L R A V 280 RM.TaqIII

RFM domain

GAT CCC TCG GTT TTC CGC GTC CAA GGC GTT GAC CCC TGG CTC TAC TTC TAC GAG GAC TTC CTG CAG GCG TAC GAC CCC GAT CTC CGC AAG GAC ATG GGG GTC TAC TAC ACC CCG GTG CCC **960** *wttaqIIRM*

GAT CCC TCG GTT TTC CGC GTC CAA GGG GTT GAC CCC TGG CTC TAC TTC TAC GAG GAC TTC CTG CAG GCG TAC GAC CCC GAT CTC CGC AGG GAC ATG GGG GTC TAC TAC ACC CCG GTG CCC 960 *wttaqIIIRM*

D P S V F R V Q G V D P W L Y F Y E D F L Q A Y D P D L R K D M G V Y Y T P V P 320 RM.TaqII

D P S V F R V Q G V D P W L Y F Y E D F L Q A Y D P D L R R D M G V Y Y T P V P 320 RM.TaqIII

Motif I: S-adenosylmethionine-binding site

GTG GTC AGG GCC ATG GTG CGC TTG GTG GAC GAG GCG CTG AAA GAG GGC TTC GGC CTG GCG GAG GGC CTG GCC CAC GAG AAG GTC ACC GTC CTG GAC CCC GCC ATG GGC ACG GGG ACC TTT **1080** *wttaqIIRM*

GTG GTC AGG GCC ATG GTG CGC TTG GTG GAC GAG GCG CTG AAA GAG GGC TTC GGC CTG GCG GAG GGC CTG GCC CAC GAG AAG GTC ACC GTC CTG GAC CCC GCC ATG GGC ACG GGG ACC TTT 1080 *wttaqIIIRM*

V V R A M V R L V D E A L K E G F G L A E G L A H E K V T V L D P A M G T G T F 360 RM.TaqII

V V R A M V R L V D E A L K E G F G L A E G L A H E K V T V L D P A M G T G T F 360 RM.TaqIII

Motif II

CTC CTC GCC ACC TTG GAA CGG GCG CTC GCA AAC ATG GCT TCC CTC TAC GGG AGG GGC TAC CGG GGA CAG TAC GCG AAA GAA GTG GCC TCC CGC CTC CAC GGC ATA GAG CTC ATG GTG GGC **1200** *wttaqIIRM*

CTC CTC GCC ACC TTG GAA CGG GCG CTC GCG AAC GTG GCC AGC CTC TAC GGG GAG GGT TAC CGG GGA CAG TAC GCG AAA GAA GTG GCC TCC CGC CTC CAC GGC ATA GAG CTC ATG GTG GGC 1200 *wttaqIIIRM*

L L A T L E R A L A N M A S L Y G R G Y R G Q Y A K E V A S R L H G I E L M V G 400 RM.TaqII

L L A T L E R A L A N V A S L Y G E G Y R G Q Y A K E V A S R L H G I E L M V G 400 RM.TaqIII

CCC TAC GCC GTG GCC CAG CTC CGC CTC TCC CAG GCG ATC CAA GGG GAA GGG GGC TCC CTC CCC GAG GAA GGC CTC AAC CTC TAC CTG GCC GAC ACC CTG GAG GCC CCG GAG GCC CCC CCT **1320** *wttaqIIRM*

CCC TAC GCC GTG GCC CAG CTC CGC CTC TCC CAG GCG ATC CAA GGG GAA GGG GGC TCC CTC CCC GAG GAA GGC CTC AAC CTC TAC CTG GCC GAC ACC CTG GAG GCC CCG GAG GCC CCC CCT 1320 *wttaqIIIRM*

P Y A V A Q L R L S Q A I Q G E G G S L P E E G L N L Y L A D T L E A P E A P P 440 RM.TaqII

P Y A V A Q L R L S Q A I Q G E G G S L P E E G L N L Y L A D T L E A P E A P P 440 RM.TaqIII

Motif IV: catalytic center of MTase

CTA GAG CAG GTC TTC TTC TAC GAG CGC CTG GCG GAG GAG CGC AAA AGG GCT GCG GAA CTC AAG CGG GAC AAG CCC ATC CTG GTG GTC CTG GGC AAT CCC CCC TAC GAT CGG GTA GAA GGG **1440** *wttaqIIRM*

CTA GAG CAG GTC TTC TTC TAC GAG CGC CTG GCG GAG GAG CGC AAA AGG GCT GCG GAA CTC AAG CGG GAC AAG CCC ATC CTG GTG GTC CTG GGC AAT CCC CCC TAC GAT CGG GTA GAA GGG 1140 *wttaqIIIRM*

L E Q V F F Y E R L A E E R K R A A E L K R D K P I L V V L G N P P Y D R V E G 480 RM.TaqII

L E Q V F F Y E R L A E E R K R A A E L K R D K P I L V V L G N P P Y D R V E G 480 RM.TaqIII

GAA AGC CAG GAG GAG CGC GAG AGG AAG GGG GGT TGG GTC CTG AGG GGG CCC CGG GAA CCC TAC CCC CTC ATG GAG GAC TTC CTC CGG CCG GCC AGG GAG GCG GAC CTG GGA ATA CAC CTC **1560** *wttaqIIRM*

GAA AGC CAG GAG GAG CGC GAG AGG AAG GGG GGT TGG GTC CTG AGG GGG CCC CGG GAA CCC TAC CCC CTC ATG GAG GAC TTC CTC CGG CCG GCC AGG GAG GCG GAC CTG GGA ATA CAC CTC 1560 *wttaqIIIRM*

E S Q E E R E R K G G W V L R G P R E P Y P L M E D F L R P A R E A D L G I H L 520 RM.TaqII

E S Q E E R E R K G G W V L R G P R E P Y P L M E D F L R P A R E A D L G I H L 520 RM.TaqIII

AAG AAC CTC TAC AAC CTC TAC GTC TAC TTC TGG CGC TTC GCC CTG TGG AAG GTC TTT GAG CAG GAC CCA GAA CGT GGT GGA GTC CTG TGC TTC ATC ACC CCC AGC TCC TAC CTC CAG GGT **1680** *wttaqIIRM*

AAG AAC CTC TAC AAC CTC TAC GTC TAC TTC TGG CGC TTC GCC CTG TGG AAG GTC TTT GAG CAG GAC CCA GAA CGT GGT GGA GTC CTG TGC TTC ATC ACC CCC AGC TCC TAC CTC CAG GGT 1680 *wttaqIIIRM*

K N L Y N L Y V Y F W R F A L W K V F E Q D P E R G G V L C F I T P S S Y L Q G 560 RM.TaqII

K N L Y N L Y V Y F W R F A L W K V F E Q D P E R G G V L C F I T P S S Y L Q G 560 RM.TaqIII

CCG GCC TTC GCC GGG ATG CGG GAA CAC GTC CGC CGG GTA GCG GAC CGG GTC TAC ATC CTG GAC CTG GGG GGA GAG GGC AGG GGA GCG GTG AGG GAG GAG AAC GTC TTC AAC ATC CAG ACC **1800** *wttaqIIRM*

CCG GCC TTC GCC GGG ATG CGG GAA CAC GTC CGC CGG GTA GCG GAC CGG GTC TAC ATC CTG GAC CTG GGG GGA GAG GGC AGG GGA GCG GTG AGG GAG GAG AAC GTC TTC AAC ATC CAG ACC 1800 *wttaqIIIRM*

P A F A G M R E H V R R V A D R V Y I L D L G G E G R G A V R E E N V F N I Q T 600 RM.TaqII

P A F A G M R E H V R R V A D R V Y I L D L G G E G R G A V R E E N V F N I Q T 600 RM.TaqIII

CCC GTG GCC ATC GCC CTG GTG GTG CGG CGC GGC CCC CAG GAC TCC CAA ACC CCG GCC CGG GTC TTC TAC CAC CGC CTG GCG CCC ACT ACT CGG GAG GAG AAG CTG AAG GAG CTG GAG GAG **1920** *wttaqIIRM*

CCC GTG GCC ATC GCC CTG GTG GTG CGG CGC GGC TCC CAG GAC TCC CAA ACC CCG GCC CAG GTC TTC TAC CAC CGC CTG GCG GCC ACT ACC CGG GAG GAG AAG CTG AAG GAG CTG GAG GAG 1920 *wttaqIIIRM*

P V A I A L V V R R G P Q D S Q T P A R V F Y H R L A P T T R E E K L K E L E E 640 RM.TaqII

P V A I A L V V R R G S Q D S Q T P A Q V F Y H R L A A T T R E E K L K E L E E 640 RM.TaqIII

Putative DNA specificity subunit

CTT CCC CCC CTC AAG GAC ATC CCC TTC CGG GAA GCC CCC AGG GAC TGG CAA GCC CCC TTC GTC CCC GAG GTG GGC GGG GAG TGG ACC AGG TGG CCC AAG CTC ACG GAC CTC TTC CCC TGG **2040** *wttaqIIRM*
CTT CCC CCC CTC AAG GAC ATC CCC TTC CGG GAA GCC CCC AGG GAC TGG CAA GCC CCC TTC GTC CCC GAG GCG GGC GGG GAG TGG GCC AGG TGG CCC AAG CTC ACG GAC CTC TTC CCC TGG 2040 *wttaqIIIRM*
L P P L K D I P F R E A P R D W Q A P F V P E V G G E W T R W P K L T D L F P W 680 RM.TaqII
L P P L K D I P F R E A P R D W Q A P F V P E A G G E W A R W P K L T D L F P W 680 RM.TaqIII

CAG CAC TCG GGG GTA GAG TTC AAG CGC ACC TGG CCC ATC GGG CCC ACC GAA GAG GTC CTG AAA AAG CGT TGG GAG ATC CTC CTG AAA GCC CCC CCC GGA GAA AGG CGT GCG CTC TTT CGG **2160** *wttaqIIRM*
CAG CAC TCG GGA GTA CAG TTC AAA CGC ACC TGG CCC ATC GGG CCC ACG AAG CAG GTT CTA GAG AAA AGG TGG GCC ATG CTC CTA GAG GCT CCT CCA GAG GAA AAG CCC CGC CTC TTC CGG 2160 *wttaqIIIRM*
Q H S G V E F K R T W P I G P T E E V L K K R W E I L L K A P P G E R R A L F R 720 RM.TaqII
Q H S G V Q F K R T W P I G P T K Q V L E K R W A M L L E A P P E E K P R L F R 720 RM.TaqIII

GAA ACA GGA GAC CGC CTA TTA GTA AAG AGT CAT CGG GCC ATC TTT AGT TCC AAG CCC TTG CCC CCC ATC GCC ACC CTT GGT CCT GGG AAC CCG CCC GAG GCC ATC GTG CGC TAC GGC TAC **2280** *wttaqIIRM*
GAA GAG CGA GAT CGG AAA GTG AGC CGG GAG TAC CGG GGA ATA TGG AGC CCG GCT TGT CTA CCT TCC CTA GAA AGC CTC ACC TCT GGG AAA CCC CCA GAA GCC ATC GTG CGC TAC GGC TAC 2280 *wttaqIIIRM*
E T G D R L L V K S H R A I F S S K P L P P I A T L G P G N P P E A I V R Y G Y 760 RM.TaqII
E E R D R K V S R E Y R G I W S P A C L P S L E S L T S G K P P E A I V R Y G Y 760 RM.TaqIII

CGG AGC TTT GAC CGC GCC TGG GCC ATC GCG GAC GGG AGG GTG TGT AGC CGT CCT CGC CCC TCC TTG TGG CAG ACC TGG AGC GAG AGG CAG GTC TAC CTC ACC TCC CTC CTC ACC ACC CCT **2400** *wttaqIIRM*
CGG AGC TTT GAC CGC GCC TGG GCC ATC GCA GAC GGG AGG GTG TGT AGC TAC CCT CGT CCC TCC TTG TGG CAG ACC TGG AGC GAG AGG CAG GTC TAC CTC ACC TCC CTC CTC ACC GCC CCC 2400 *wttaqIIIRM*
R S F D R A W A I A D G R V C S R P R P S L W Q T W S E R Q V Y L T S L L T T P 800 RM.TaqII
R S F D R A W A I A D G R V C S Y P R P S L W Q T W S E R Q V Y L T S L L T A P 800 RM.TaqIII

CTA GGA AGA GGA CCT GCC CTG GTG GCC ACG GCC TAC CTT CCT GAC TTG CAC CAC TTC AGT — TCC CGT GGA GGC AAG GAC ATC ATC CCC CTC TTC CGC GAC CGG GAG GGC CGG GAG CCC **2517** *wttaqIIRM*
CTA GGG AGA GGA CCT GCC CTG GTG GCC ACG GCC TGC GTT CCT GAC TTG CAC CAC TTT AGG GGC TCC TTC GGC GGG AAG GAT GTC ATC CCC CTC TTC CGC GAC CGG GAG GGC CGG GAG CCC 2520 *wttaqIIIRM*
L G R G P A L V A T A Y L P D L H H F S — S R G G K D I I P L F R D R E G R E P 839 RM.TaqII
L G R G P A L V A T A C V P D L H H F R G S F G G K D V I P L F R D R E G R E P 840 RM.TaqIII

AAC CTG ACA CGG GGC CTC CTG AAG CTC CTG GAA GAG GCC TAC GGC TTT CCC GTC TCC CCC GAG GAC TTC GCC GCC TAC GTC TAC GCC CTC CTG GCC CAC CCC GCC TAC ACG GAA CGC TTC **2637** *wttaqIIRM*
AAC CTA ACA CGG GGC CTC CTG AAG CTC CTG GAA GCG GCC TAC GGC GTT CCC GTC TCC CCC CAG GAC TTC GCC GCC TAC GTC TAC GCC CTC CTG GCC CAC CCC GCC TAC ACG GAA CGC TTC 2640 *wttaqIIIRM*
N L T R G L L K L L E E A Y G F P V S P E D F A A Y V Y A L L A H P A Y T E R F 879 RM.TaqII
N L T R G L L K L L E A A Y G V P V S P Q D F A A Y V Y A L L A H P A Y T E R F 880 RM.TaqIII

GCG GAA GAG CTC AGG GTA CCT GGG CCC AGG GTC CCG CTC ACC AAG GAC CCC AGC CTC TTC AGG GAG GGC GCG GAG CTG GGG GCC TAC CTC CTC TGG CTC CAC ACC TAC GGG GAA CGG TAC **2757** *wttaqIIRM*
GCG GAA GAG CTC AGG GTA CCC GGG CCC AGG GCC CCC ATT ACC AAG GAC CCC AGC CTC TTC AGG GAG GGC GTG GAG CTG GGG GCC CAC CTC CTC TGG CTC CAC ACC TAC GGG GAA CGG TAC 2760 *wttaqIIIRM*
A E E L R V P G P R V P L T K D P S L F R E G A E L G A Y L L W L H T Y G E R Y 919 RM.TaqII
A E E L R V P G P R A P I T K D P S L F R E G V E L G A H L L W L H T Y G E R Y 920 RM.TaqIII

GCC GAG GGG CGG AGC TGG CCC CCC AAG GGC AGG GCC CGC TGG GCC AAA CCC CCC TCC GCC TAC CCC GAG GGG CAC AGC TAC GAC CCC GAA ACG AGA ATC CTT CAC GTG GGA GAC GGG GAG **2877** *wttaqIIRM*
GCC GAG GGG CGA AGC TGG CCC CCC AAG GGC AGA GCC CGT TGG GCC AAA CCC CCC TCC GCC TAC CCC GAG GGG CAC AGC TAC GAC CCC GAG ACG AGA ATC CTT CAC GTG GGA GAC GGG GAG 2880 *wttaqIIIRM*
A E G R S W P P K G R A R W A K P P S A Y P E G H S Y D P E T R I L H V G D G E 959 RM.TaqII
A E G R S W P P K G R A R W A K P P S A Y P E G H S Y D P E T R I L H V G D G E 960 RM.TaqIII

GTA GAG GAC GTA GCC CCA GAG GTC TAC GGG TTT GAG GTC TCC GGC TTC CTT CCA GTA GAG AGC TGG CTG GGG TTC CGC CAG AGG AAC AGG AGG GGG CGG AGG AGT AGC CCT TTG GAT GAT **2997** *wttaqIIRM*

GTA GAG GAC GTA GCC CCA GAG GTC TAC GGG TTT GAG GTC TCC GGC TTC CTT CCA GTA GAG AGC TGG CTG GGG TTC CGC CAG AAG AAC AGG AGG GGG CGG AGG AGT AGC CCT TTG GAT GAT 3000 *wttaqIIIRM*

V E D V A P E V Y G F E V S G F L P V E S W L G F R Q R N R R G R R S S P L D D 999 RM.TaqII

V E D V A P E V Y G F E V S G F L P V E S W L G F R Q K N R R G R R S S P L D D 1000 RM.TaqIII

GTC GTC CCT TCG GAA TGG CCC GCA GAC TTA GGC CGG GAG CTT CTG GAA CTC CTC TGG GTC TTG GAG AAG ACC CTG GAG ATC TAC CCT GAG CAG AAG GAG CTT CTG CAA AAG GTT CTG GAG **3117** *wttaqIIRM*

GTC GTC CCT TCG GAA TGG CCC GCA GAC TTA GGC CGG GAG CTT CTG GAA CTC CTC TGG GTC TTG GAG AAG ACC CTG GAG ATC TAC CCT GAG CAG AAG GAG CTT CTG CAA AAG GTT TTG GAG 3120 *wttaqIIIRM*

V V P S E W P A D L G R E L L E L L W V L E K T L E I Y P E Q K E L L Q K V L E 1039 RM.TaqII

V V P S E W P A D L G R E L L E L L W V L E K T L E I Y P E Q K E L L Q K V L E 1040 RM.TaqIII

GGC CCC CTC TTT ACC GTG GAT GAA CTT CCG ACC CCG ACT CCT GAA CAG CGG GAA CCG CCT GGG GGA GAA GAG GAG AAA CCC CAG GAA GCT GAA GCG GTT GGA GAG GAG GAG GGA GAG AAC **3237** *wttaqIIRM*

GGC CCC CTC TTT ACC GTG GAT GAG CTT CCG GCC CCG ACT CCC GAA CAG CGG CGG GAG CCT GGG GGA AAA GAG GAG GAA CCT CAA GAA GTT GAG GCG GTT GGA GAG GAG GAG GGA GAG AAC 3240 *wttaqIIIRM*

G P L F T V D E L P T P T P E Q R E P P G G E E E K P Q E A E A V G E E E G E N 1079 RM.TaqII

G P L F T V D E L P A P T P E Q R R E P G G K E E E P Q E V E A V G E E E G E N 1080 RM.TaqIII

GGC GCG GAG CAC GTG GTC CAG CCC AGG CTC CTC TCC CTC AGA GAA GCG AGT AGG GAC GGA GTC TAC GGG AAC CAA CCG *TGA* **3318** *wttaqIIRM*

GGC GCG GAG CGC GTG GTC CAA CCC AGG CTC TTC TCT CCC GGG GGA AGA AAC AGG GAC GGG GTC *TAG* 3306 *wttaqIIIRM*

G A E H V V Q P R L L S L R E A S R D G V Y G N Q P * 1105 RM.TaqII

G A E R V V Q P R L F S P G G R N R D G V * 1101 RM.TaqIII

Figure S5. Comparison of the native TaqII and TaqIII nt and aa sequences. The predicted aa sequences of the proteins are indicated in capital letters. The DNA sequence of the *taqIIRM* is written in black. The DNA sequence of the *taqIIIRM* is written in blue. The changed bases are marked in red. The crucial aa of the catalytic centres are dark red, bold and underlined. The functional protein domains are marked as follows: REase domain in blue, helical domain in light green, MTase domain in dark green and the potential TRD region in brown. Numbering of nt of the gene variants and polypeptide aa starts as ‘1’ with the beginning (ATG) of *taqIIRM* and *taqIIIRM* ORFs. Differences in aa sequences between TaqII and TaqIII are marked in yellow.

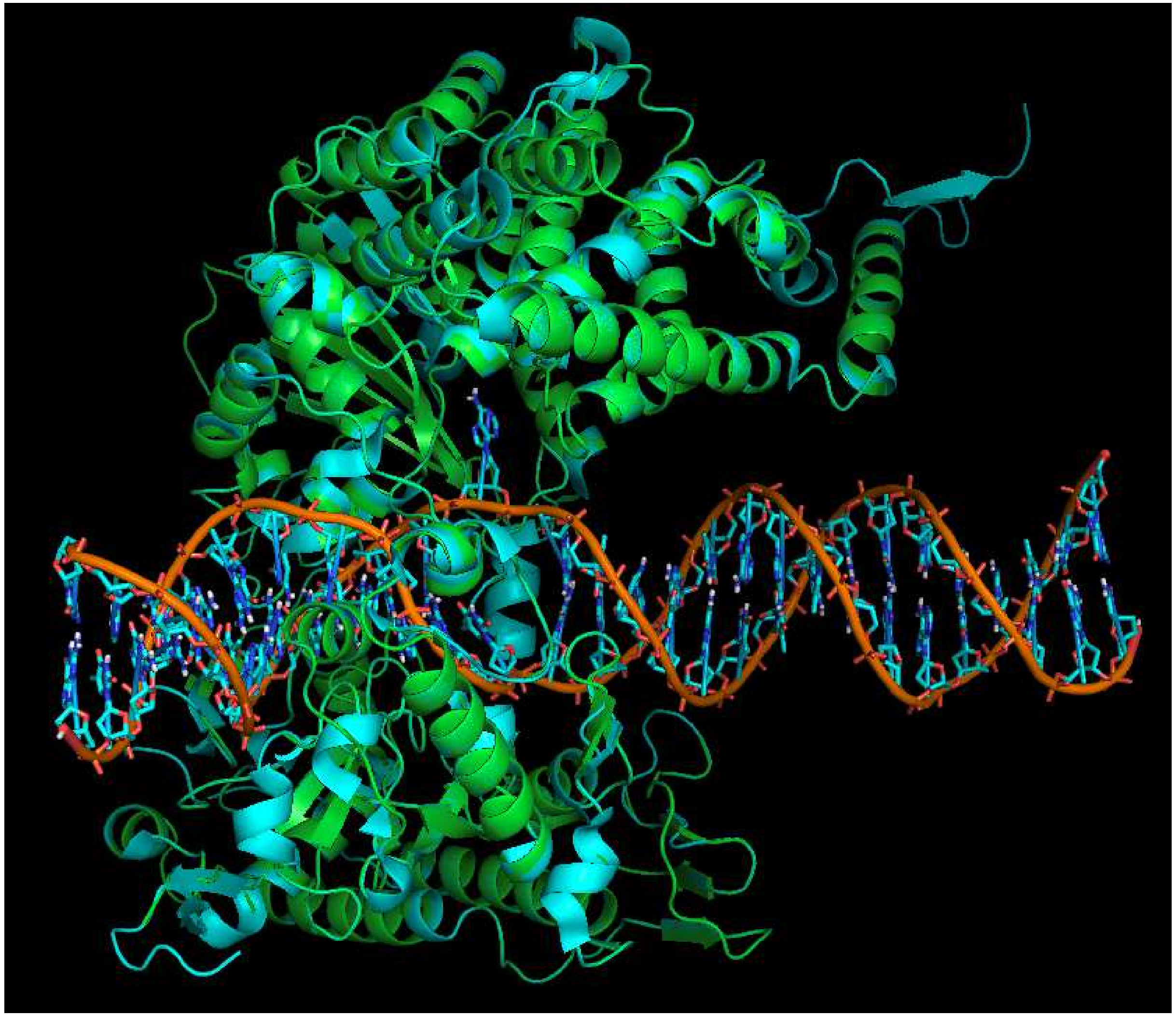

Figure S6. Similarity between predicted TaqII and actual LlaBIII structure. A model of TaqII structure was built by the PHYRE server (http://www.sbg.bio.ic.ac.uk/phyre2/html/page.cgi?id=index), using the LlaBIII structure as template. The image is a cartoon depiction of the protein and DNA, with LlaBIII in cyan and TaqII in green. The adenine that is methylated is flipped out into the MTase domain. The DNA is from the LlaBIII structure.

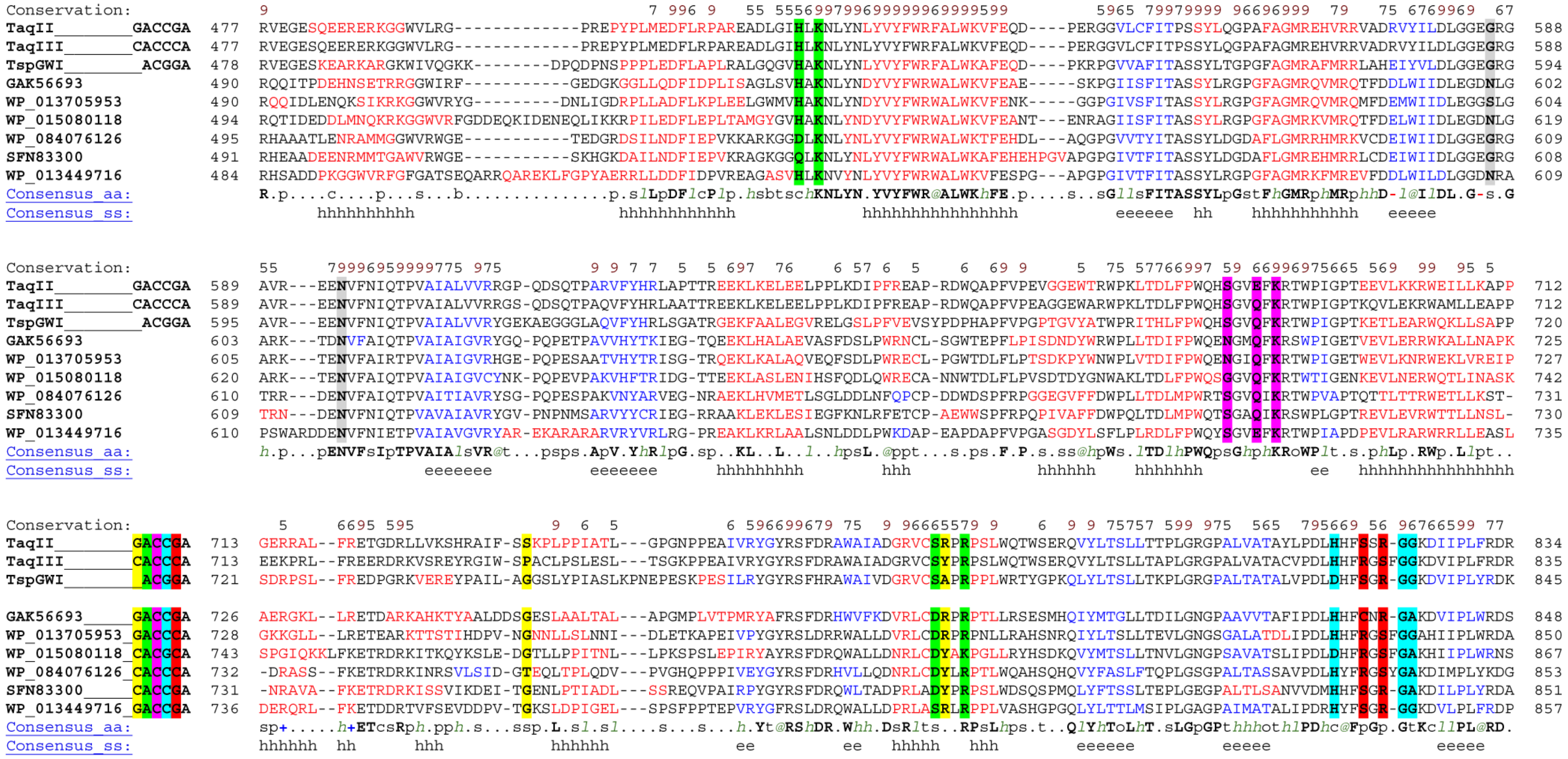

Figure S7. Multiple sequence alignment of the characterized *Thermus*-family protein sequences and close homologs (Table 2). Putative DNA recognition elements are highlighted. Predictions for the recognition specificity of the uncharacterized homologs (bottom panel) are given, based on the similarity of their recognition elements to characterized *Thermus*-family and Type ISP enzymes.

Sequence alignments were performed using PROMALS3D. 'Consensus aa' indicates consensus amino acid residues. 'Consensus ss' indicates predicted secondary structure, were 'h' indicates helix (in red), and 'e' indicates beta strand (in blue).

Table S1. Peptide sequences determined from MS-MS/MS data, unique for TaqIII protein. Aa non-matching to TaqII aa sequence are in bold and underlined.

Lp.	Aa sequence	Localization
1.	MT**E**DTWVLS**V**R	PD-(D/E)XK domain
2.	EAVLEEVRENP**K**	helical domain
3.	**R**DMGVYYTPVPVVR	helical domain
4.	ALAN**W**ASLYG**E**GYR	RMF domain
5.	RG**S**QDSQTPARVFYHR	RMF domain
6.	DWQAPFVPEVGGEW**A**R	TRD
7.	LTDLFPWQHSGV**Q**FK	TRD
8.	RTWPIGPT**K**	TRD
9.	P**TS**G**K**PPEAIVR	TRD
10.	VCS**Y**PR	TRD
11.	QVYLTSLLT**A**PLGR	TRD
12.	D**V**IPLFR	TRD
13.	EG**V**ELGA**H**	TRD
14.	VLEGPLFTVDELP**A**PTPEQR	TRD

Table S2. Putative ORFs of pAYT1_11 and their functions.

ORF Name*	Position in sequence		Protein length	Predicted gene function	Most relevant homolog		
	Strand	bp	(aa)		Numbers of identities/ number examined(%)	Organism (plasmid)	GenBank accesion number
ORF 1	+	340-666	108	AsnC family transcriptional regulator	25/76(33)	*Tateyamaria sp.* ANG-S1	WP039689333.1
ORF 2	+	789-2777	662	Transcriptional regulator	396/659 (60)	*Thermus oshimai*	WP038036902.1
ORF 3	+	3272-5572	766	Hypothetical protein	630/750(84)	*Thermus oshimai*	WP018461242.1
parA	+	5676-6278	200	chromosome partitioning protein ParA	183/200(92)	*Thermus oshimai*	WP026234140.1
copG	+	6275-6502	75	CopG family transcriptional regulator	26/80(33)	*Nostoc sp.* MBR 210	WP044501083.1
taqIIIRM	+	6537-9842	1101	Type IIC/IIG REase	1028/1101(93)	*Thermus aquaticus*	AAL23675.1
ORF 7	+	10127-10579	150	Cell wall binding protein	61/122 (50)	*Paenibacillus algorifonticola*	WP052737010.1

*Bioinformatic analysis predicted 18 open reading frames (ORFs). The predicted ORFs without assigned biochemical function were removed for clarity.

Table S3. Putative ORFs of pAYT1_61 and their functions.

ORF Name*	Position in sequence		Protein length	Predicted gene function	Most relevant homolog		
	Strand	bp	(aa)		Numbers of identities/ number examined (%)	Organism (plasmid)	GenBank accession number
ORF 1	+	7,187-7,660	157	TrbC/VIRB2 family protein	131/131(100)	*Thermus aquaticus*	KOX88964.1
ORF 2	+	7,966-10,509	847	conjugal transfer protein TraC	842/847(99)	*Thermus aquaticus*	WP_003049213.1
ORF 3	+	14,244-16,688	814	type IV secretion system protein VirD4	814/814(100)	*Thermus aquaticus*	WP_053768696.1
ORF 4	+	17,183-17,638	151	lytic transglycosylase	151/151(100)	*Thermus aquaticus*	WP_053768698.1
ORF 5	+	17,956-18,915	319	AAA family ATPase	319/319(100)	*Thermus aquaticus*	WP_053768700.1
ORF 6	+	18,981-20,396	471	S-layer protein	471/471(100)	*Thermus aquaticus*	WP_053768701.1
ORF 7	+	23,214-23,903	229	DNA-binding protein	229/229(100)	*Thermus aquaticus*	WP_053768705.1
ORF 8	+	24,420-25,013	197	peptidase	197/197(100)	*Thermus aquaticus*	WP_053768707.1
ORF 9	+	26,359-27,117	252	RNA polymerase subunit sigma-70	251/252(99)	*Thermus aquaticus*	WP_053768710.1
ORF 10	+	34,250-35,332	360	transposase IS4 family protein	360/360(100)	*Thermus aquaticus* Y51MC23	EED09614.1
ORF 11	+	40,566-41,606	346	hypothetical protein TaqDRAFT_3380	301/347(87)	*Thermus aquaticus* Y51MC23	EED09085.1

ORF 12	+	45,168-46,520	450	peptidase S8 and S53 subtilisin kexin sedolisin	337/386(87)	*Thermus aquaticus* Y51MC23	EED09079.1
ORF 13	+	47,246-48,031	261	tyrosine recombinase XerC	261/261(100)	*Thermus aquaticus*	KOX88944.1
parA	+	49,289-50,260	323	chromosome partitioning protein ParA	323/323(100)	*Thermus aquaticus*	WP_053768678.1
parB	+	50,512-51,147	211	chromosome partitioning protein ParB	211/211(100)	*Thermus aquaticus*	WP_053768679.1
taqIIRM	+	51,144-54,461	1105	TaqII<br>Type IIC/IIG REase	1104/1105(99)	*Thermus aquaticus*	AAL23675.1
ORF 17	-	56,943-55,858	361	helix-turn-helix domain-containing protein	176/362(49)	*Thermus aquaticus*	WP_053766901.1
ORF 18	-	57,852-57,208	214	transcriptional regulator	214/214(100)	*Thermus aquaticus*	WP_053768681.1

*Bioinformatic analysis predicted 125 ORFs: 43 ORFs annotated as hypothetical genes and 18 with assigned function. All the ORFs without assigned biochemical function were removed for clarity.

Table S4. Differences between TaqII and TaqIII polypeptide sequences. Localization of aa substitutions.

**No.**	**aa substitution** (X_yZ) [TaqIII →TaqII]	**localization**	**aa substitutions**
1.	$E_{3}G$, $V_{10}I$, $Q_{125}E$	REase catalytic domain	3
2.	$K_{186}N$, $R_{310}K$	MTase helical domain	2
3.	$V_{372}M$, $E_{378}R$, $S_{612}P$, $Q_{620}R$, $A_{628}P$	MTase catalytic domain	5
4.	$A_{664}V$, $A_{669}T$, $Q_{686}E$, $K_{697}E$, $Q_{698}E$, $E_{701}K$, $A_{705}E$, $M_{706}I$, $E_{709}K$, $E_{713}G$, $K_{715}R$, $P_{716}R$, $R_{717}A$, $E_{722}T$, $R_{723}G$, $K_{726}L$, $V_{727}L$, $S_{728}V$, $R_{729}K$, $E_{730}S$, $Y_{731}H$, $G_{733}A$, $W_{735}F$, $P_{737}S$, $A_{738}K$, $C_{739}P$, $S_{742}P$, $L_{743}I$, $E_{744}A$, $S_{745}T$, $T_{747}G$, $S_{748}P$, $K_{750}N$, $Y_{777}R$, $A_{799}T$, $C_{812}Y$, $V_{813}L$, $R_{820}S$, $F_{823}R$, $V_{828}I$, $A_{852}E$, $V_{856}F$, $Q_{861}E$, $A_{891}V$, $I_{893}L$, $V_{904}A$, $H_{909}Y$, $K_{987}R$, $A_{1051}T$, $R_{1058}E$, $E_{1059}P$, $K_{1063}E$, $E_{1066}K$, $V_{1070}A$, $R_{1084}H$, $F_{1091}L$, $P_{1093}L$, $G_{1094}R$, $G_{1095}E$, $R_{1096}A$, $N_{1097}S$	Putative DNA specificity subunit	61
